# EINN: An enzyme-informed neural network guided by an enzyme-constrained genome-scale metabolic model

**DOI:** 10.1016/j.synbio.2026.07.009

**Published:** 2026-08-06

**Authors:** Ray Steven, Kai Kimata, Denis Chegodaev, Lilies Handayani, Kenji Satou

**Affiliations:** a Graduate School of Natural Science and Technology, Kanazawa University, Kanazawa, 9201192, Japan; b Faculty of Transdisciplinary Sciences for Innovation, Institute of Transdisciplinary Science for Innovation, Kanazawa University, Kanazawa, 9201192, Japan

**Keywords:** Hybrid model, Machine learning, Multi-omics, Genome-scale metabolic model, Neural networks

## Abstract

Predicting cellular metabolism from molecular data is a central challenge in systems biology, with direct implications for engineering microbial cell factories. Genome-scale metabolic models (GEMs) provide a mechanistic foundation for such predictions, but their accuracy is limited by an inability to account for the finite catalytic capacity of the proteome. Enzyme-constrained GEMs (ecGEMs) address this by incorporating enzyme turnover numbers and proteome allocation constraints. Hybrid neural-mechanistic models like Artificial Metabolic Network (AMN) and Metabolic-Informed Neural Network (MINN) have sought to combine the flexibility of machine learning with the structure of GEMs, yet they rely on conventional, unconstrained metabolic networks, allowing flux predictions to violate enzyme capacity constraints and pushing models toward biologically unrealistic solution spaces. Here, we introduce the Enzyme-Informed Neural Network (EINN), a conceptual framework which relies on ecGEMs to constrain the neural network. We implement two variants of EINN, ecAMN and ecMINN, using *Escherichia coli* (*E. coli*) ecGEMs built with the GECKO 3.0 toolbox, and systematically evaluate their performance. Compared to their unconstrained counterparts, ecAMN significantly improves training stability and predictive accuracy by eliminating convergence to poor local minima, while ecMINN reduces overfitting and achieves lower error rates through mechanistic integration of proteomic data as flux bounds. Together, these results show that constraining the neural network's solution space with enzymatic capacity limits is a more effective hybrid modeling foundation than relying on conventional GEMs alone.

## 1 Introduction

Genome-scale metabolic models (GEMs) are foundational computational representations that have been used for decades to mathematically explore the metabolic behaviors of microorganisms and other organisms [1,2]. Their broad application in biotechnology, biomedicine, and fundamental studies has accelerated our ability to predict cellular phenotypes under diverse environmental conditions [3]. These models, built from an organism's full complement of metabolic reactions, are typically solved using constraint-based methods such as Flux Balance Analysis (FBA) to identify feasible metabolic states [4].

Despite their widespread utility, the predictive scope of conventional GEMs remains limited, and many phenotypes cannot be quantitatively captured [5]. A primary challenge lies in achieving accurate quantitative predictions, which often require labor-intensive experimental measurements of nutrient uptake fluxes to properly constrain the model for each specific condition [6]. Furthermore, conventional GEMs do not account for the finite catalytic capacity of enzymes that drive metabolism [7]. This omission prevents them from capturing essential resource-allocation phenomena, such as overflow metabolism, thereby restricting their biological realism and predictive power [8]. These limitations underscore the need for models that more tightly integrate mechanistic constraints with data-driven learning.

To address the absence of enzymatic realism in conventional GEMs, several extensions have been developed to incorporate catalytic capacity directly into model structure. Among these, the GECKO (Genome-scale metabolic model with Enzymatic Constraints using Kinetic and Omics data) framework has been widely adopted to construct more realistic enzyme-constrained models (ecModels) for a diverse range of organisms [9,10]. The GECKO method is founded on the principle that the rate of any enzymatic reaction is limited by the abundance of the enzyme that catalyzes it and its maximal turnover number (*k*_cat_) [10,11]. Each enzyme is thus incorporated as a pseudo-substrate into its catalyzed reaction, drawing from a shared protein pool representing the total mass of available enzymes [10,12]. By introducing this additional layer of constraint, ecModels substantially reduce the feasible solution space and can capture complex phenotypes such as overflow metabolism and proteome allocation, which are typically beyond the reach of conventional GEMs [7,9,13].

In parallel, hybrid modeling approaches that integrate machine learning (ML) with mechanistic representations have emerged as powerful strategies to enhance predictive capability. The Artificial Metabolic Network (AMN) framework exemplifies this direction as a neural-mechanistic hybrid model that embeds a constraint-based metabolic network directly within a differentiable neural architecture [14]. Unlike earlier approaches [15] where ML was used as a pre-processing [16,17] or post-processing layer [[18], [19], [20]], AMNs make the FBA computation itself part of the learning process by replacing the traditional, non-differentiable simplex solver with custom differentiable solvers. This allows end-to-end gradient propagation during training, enabling the model to learn complex relationships directly from data, such as predicting nutrient uptake fluxes from medium composition, while maintaining mechanistic consistency. Similarly, the Metabolic-Informed Neural Network (MINN) further integrates multi omics features to improve generalization and biological interpretability [21].

**Fig. 1:**
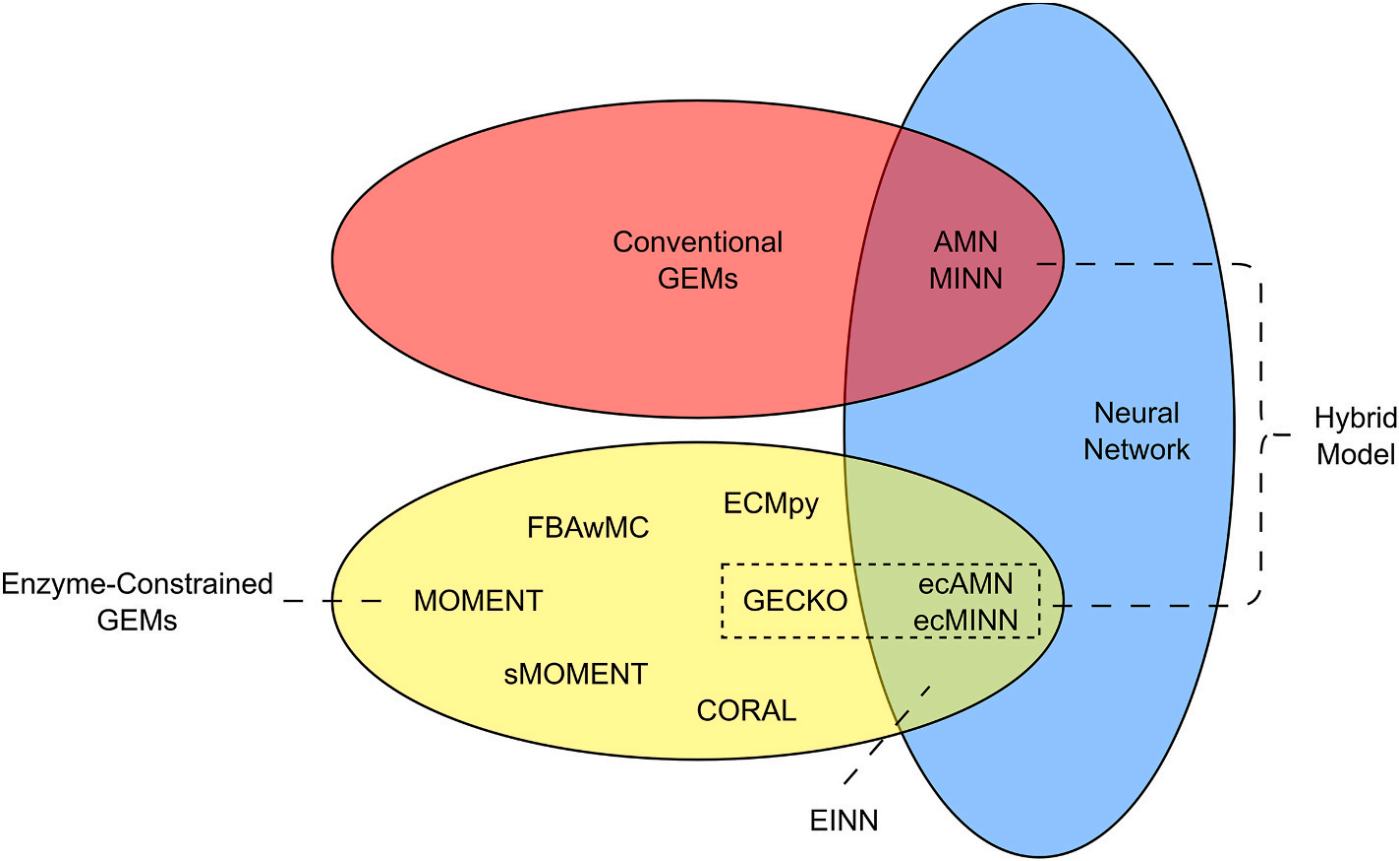
**Conceptual overview of the EINN framework.** The EINN framework creates a direct bridge between ecGEMs and neural network architectures. Current ecGEM tools—including GECKO [10], CORAL [22], sMOMENT [13], MOMENT [23], ECMpy [24], and FBAwMC [25]—provide mechanistic models with explicit enzyme capacity and proteome-allocation constraints, yet remain separate from neural approaches. In contrast, existing hybrid neural–mechanistic models such as AMN [14] and MINN [21] rely on conventional GEMs and therefore lack enzyme-aware constraints. EINN integrates these domains by embedding ecGEMs within neural networks, enabling enzyme-informed hybrid models that combine mechanistic enzyme limitations with data-driven learning. In this research we implemented a specific use case of EINN that uses the GECKO method (dashed line).

Although these hybrid models effectively combine data-driven and mechanistic reasoning, their metabolic layer still relies on conventional GEMs that lack enzymatic and proteomic constraints. Consequently, their predictions, while accurate, may not fully respect the biochemical limitations imposed by enzyme availability and catalytic efficiency. Integrating enzyme-constrained models into such hybrid frameworks could thus enrich the learning process with more biological grounding. Here, we introduce the Enzyme-Informed Neural Network (EINN) framework (Fig. 1), a conceptual approach that extends existing hybrid modeling architectures by embedding enzyme-constrained genome-scale models (ecGEMs) within their mechanistic cores. This integration brings together the mechanistic physiological realism of enzyme-constrained models and the adaptive learning capacity of neural architectures. Within this conceptual framework, we implement two specific variants, ecAMN and ecMINN, which embed ecGEMs (built using GECKO method) into the AMN and MINN architectures, respectively. We showed that by enforcing enzymatic feasibility during learning yield predictions that are not only more accurate but also increase training stability compared to hybrid models that are based solely on unconstrained GEMs.

## 2 Methods

### 2.1 Conceptual framework

**Fig. 2:**
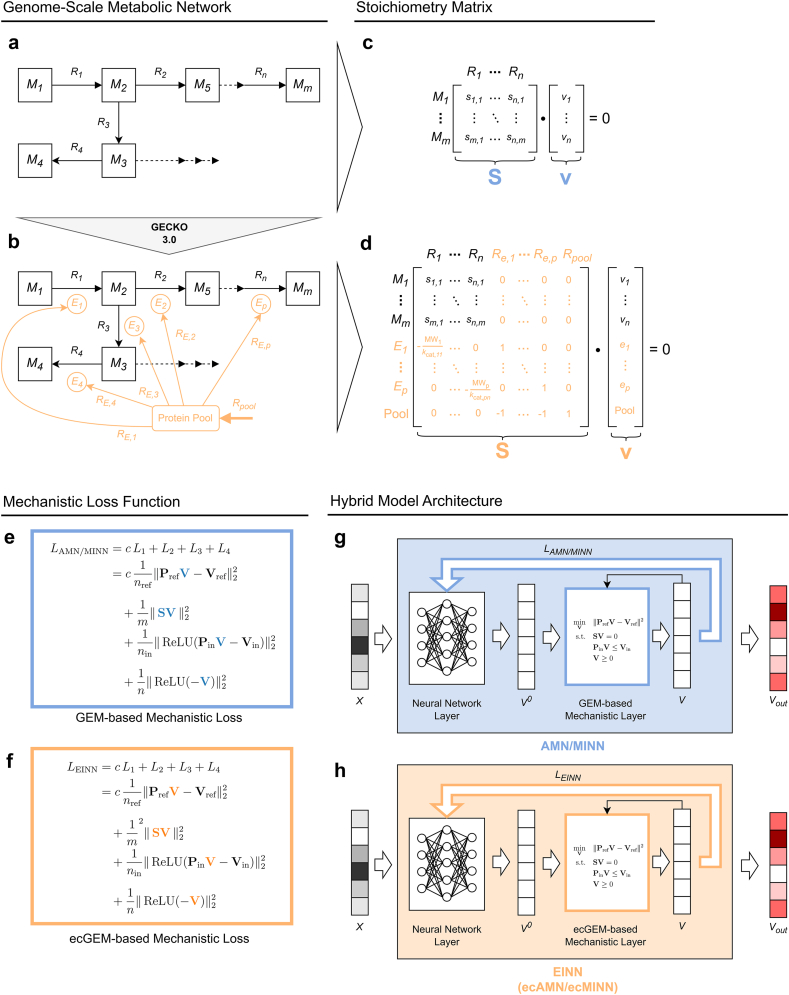
**Schematic overview of the EINN framework. a,b** Conventional GEM and ecGEM structures, illustrating how GECKO 3.0 expands the network with enzyme usage and protein-pool constraints. **c,d** Corresponding stoichiometric matrices for GEMs and ecGEMs, showing the introduction of enzyme pseudo-metabolites and protein-pool reactions. **e,f** Mechanistic loss functions used in AMN/MINN (GEM-based) and EINN (ecGEM-based), where constraint violations are penalized during training. **g,h** Hybrid neural-mechanistic architectures integrating a neural network layer with a GEM or ecGEM mechanistic layer. EINN extends AMN/MINN by embedding ecGEM constraints, enabling flux predictions that satisfy steady-state mass balance and enzyme capacity limits.

To overcome the limitations of current hybrid models regarding the finite catalytic capacity of the proteome, we proposed the EINN framework (Fig. 1). The framework operates by utilizing ecGEMs as constraints for neural networks. While traditional hybrid models rely on the conventional metabolic network structure (Fig. 2a), the EINN framework utilizes the enzyme-expanded network structure (Fig. 2b). This structural difference dictates how the model enforces constraints during the learning process. Standard models and hybrid models (AMN and MINN) that rely on conventional GEMs, constrain predictions using the basic stoichiometric matrix (Fig. 2c). In contrast, EINN framework models project predictions onto a solution space defined by the expanded stoichiometric matrix of ecGEM (Fig. 2d) which explicitly accounts for enzyme usage and total protein pool availability.

This architectural shift is mathematically enforced by redefining the mechanistic constraints within the loss function rather than altering its fundamental structure. As illustrated in the comparison between the standard GEM-based loss (Fig. 2e) and the ecGEM-based loss (Fig. 2f), the formulation remains structurally consistent. However, the stoichiometric violation term (***SV***) in the EINN framework is intrinsically expanded to penalize violations of enzyme capacity and protein availability alongside mass balance. This formulation forces the neural network to navigate a solution space that is economically feasible for the cell rather than just chemically balanced. We implemented two specific variants to evaluate this framework, specifically ecAMN and ecMINN, to allow for a direct benchmarking of performance improvements attributable to these enzyme constraints. The distinction between the standard hybrid architecture and our proposed EINN framework is illustrated in the architectural diagrams (Fig. 2g and h).

### 2.2 Model architecture and formulation

#### 2.2.1 Neural network layer

The EINN adopts the two-part hybrid structure established in AMN and MINN: a data-driven neural layer followed by a mechanistic (ecGEM) layer (Fig. 2g and h). The neural layer is a fully connected feed-forward network that maps experimental features (***X***) such as multi-omics profiles and/or media conditions (***V***_in_) to an initial estimate of metabolic fluxes (***V***^0^):

(1) $$V^{0}=NN\left(X;\theta_{\mathrm{NN}}\right)$$

where *θ*_NN_ represents the trainable parameters of the neural network. This layer captures complex nonlinear relationships between environmental or molecular states and metabolic activity, learning implicit regulatory signals not explicitly encoded in the mechanistic model. The mechanistic layer refines the neural prediction by projecting ***V***^0^ onto the feasible solution space of an ecGEM. It iteratively adjusts fluxes through a differentiable, gradient-based procedure to produce the final, feasible flux distribution ***V***_out_ compliance with the enzymatic constraints of the ecGEM.

#### 2.2.2 Enzyme-constrained mechanistic core

The mechanistic core of EINN is based on enzyme-constrained GEM, which expands the conventional steady-state formulation of genome-scale models to include enzyme capacity constraints following the GECKO 3.0 [26] framework for the Stage 3 ecGEM (Fig. 2a–d). The constraint system is defined as:

(2) $$\begin{array}{c} Sv=0\\ a_{i}\leq v_{i}\leq b_{i}\\ \begin{array}{c} -\frac{MW_{j}}{k_{\mathrm{cat},ji}}v_{i}-e_{j}=0\\ \sum_{j}e_{j}-pool=0\\ \begin{array}{c} -1000\leq e_{j}\leq 0\\ -\sigma fP_{\mathrm{tot}}\leq pool\leq 0 \end{array} \end{array} \end{array}$$

Here, ***S*** is the expanded stoichiometric matrix (S-matrix), which includes not only metabolite mass balances but also pseudo-metabolites representing enzyme usages and the total protein pool, as introduced in the GECKO 3.0 framework and **v** is the flux vector (Fig. 2d). Each reaction flux is denoted by *v*_*i*_, with lower and upper bounds *a*_*i*_ and *b*_*i*_, respectively. The term *MW*_*j*_ represents the molecular weight of enzyme *j*, and *k*_cat,*ji*_​ is the catalytic turnover number of enzyme *j* for reaction *i*. The variable *e*_*j*_ corresponds to the usage of enzyme *j*, expressed in mmol gDW^−1^, while *pool* represents the total available proteome fraction allocated to metabolism. The parameters *σ* and *f* denote, respectively, the average enzyme saturation and the fraction of the total proteome dedicated to metabolic enzymes, and *P*_tot_ is the total cellular protein content (g protein gDW^−1^). All these constraints are integrated into the structure of ***S***, allowing EINN to enforce enzymatic capacity within the same mathematical framework used for stoichiometric mass balance (Fig. 2e–h).

#### 2.2.3 Quadratic programming formulation for mechanistic layer

Following the formulation proposed in the AMN framework, the mechanistic layer aims to find a flux vector ***V*** that best reproduces experimentally observed fluxes while satisfying all biochemical constraints. This objective can be expressed as a quadratic programming (QP) problem:

(3) $$\begin{array}{c} \min_{V}\;{\left\|P_{\mathrm{ref}}V-V_{\mathrm{ref}}\right\|}^{2}\\ s.\;t.\;SV=0\\ \begin{array}{c} P_{\mathrm{in}}V\leq V_{\mathrm{in}}\\ V\geq 0 \end{array} \end{array}$$

In this formulation, ***V*** is the vector of all reaction fluxes predicted by the model; ***S*** is the expanded stoichiometric matrix as described above; ***P***_ref_ and ***V***_ref_ denote the projection operator and the corresponding reference fluxes used for supervision; and ***P***_in_ and ***V***_in_ correspond to uptake or input constraints defining the boundary conditions of the system. The non-negativity constraint ***V*** ≥ 0 enforces directionality in irreversible reactions. This QP formulation defines the optimal feasible flux distribution consistent with both experimental data and the enzyme-constrained metabolic network.

#### 2.2.4 Loss function derivation

To enable end-to-end differentiable training, the QP formulation above is approximated by a differentiable composite loss function, following the AMN structure and incorporating the weighting coefficient *c* from the MINN framework for ecMINN implementation. The total training objective is defined as:

(4) $$L_{\mathrm{EINN}}=cL_{1}+L_{2}+L_{3}+L_{4}=c\frac{1}{n_{\mathrm{ref}}}{\left\|P_{\mathrm{ref}}V-V_{\mathrm{ref}}\right\|}_{2}^{2}+\frac{1}{m^{2}}{\left\|SV\right\|}_{2}^{2}+\frac{1}{n_{\mathrm{in}}}{\left\|\mathrm{ReLU}\left(P_{\mathrm{in}}V-V_{\mathrm{in}}\right)\right\|}_{2}^{2}+\frac{1}{n}{\left\|\mathrm{ReLU}\left(-V\right)\right\|}_{2}^{2}$$

In this expression, ***V*** (or ***V***_out_) denotes the full predicted flux vector. The scalar *c* weights the data-driven term relative to the mechanistic terms (as in MINN). ***P***_ref_ is the projection operator that selects the subset of fluxes for which experimental reference values ***V***_ref_ are available, and *n*_ref_ is the number of those reference fluxes. ***S*** is the expanded stoichiometric matrix (including enzyme pseudo-metabolites and the protein pool); *m* denotes the number of rows of ***S*** (metabolites and pseudo-metabolites). We divide the stoichiometric penalty by *m*^2^ to improve numerical stability and scale the magnitude of the *L*_2_ term appropriately for large GEMs such as ecGEMs, where ∥***SV***∥_2_ can otherwise dominate the objective. ***P***_in_ projects ***V*** onto the subset of input/uptake fluxes with known bounds ***V***_in_, and *n*_in_ is the number of such input-bounded fluxes. Finally, *n* is the total number of flux variables in ***V***. The ReLU- function implements one-sided penalties so that *L*_3_ penalizes predicted inputs that exceed their known upper bounds (***P***_in_***V*** > ***V***_in_) and *L*_4_ penalizes negative flux values where negativity is disallowed. The first term, *L*_1_, is the data-driven loss, and the sum of *L*_2_+*L*_3_+*L*_4_ forms the mechanistic loss to enforce stoichiometry, reaction bounds, and reaction bounds positivity. During training *L*_EINN_ is minimized with respect to the neural parameters *θ*_NN_.

### 2.3 Enzyme-constrained genome scale metabolic models construction and its evaluation

#### 2.3.1 ecGEM construction

The enzyme-constrained models of *E. coli* were constructed using the GECKO Toolbox version 3.0 [26] with the highly curated genome-scale models iML1515 [27] and iAF1260 [28] as scaffolds. The reconstruction followed Stages 1–3 of the GECKO 3.0 protocol and encompassed model expansion, integration of *k*_cat_, and model parameter tuning [26]. The GECKO 3.0 input parameters *σ* and *P*_tot_ were set to a default value of 0.5 while the *f* parameter was calculated using protein abundance data from PaxDB version 5.0 [29]. The *k*_cat_ values for each enzyme-substrate pair were sourced from the deep learning predictions of DLKcat [30] as integrated into the GECKO 3.0 pipeline. To ensure accurate simulation of the maximal growth rate of *E. coli*, the draft ecGEMs were further calibrated against experimental phenotypes. Specifically, we utilized the automated *k*_cat_ tuning procedure built into the default GECKO 3.0 pipeline to relieve growth-limiting bottlenecks, optimizing the model against experimental data [31].

#### 2.3.2 ecGEM evaluation

To characterize the fundamental impact of enzyme constraints on the metabolic solution space, we first employed flux sampling and Principal Component Analysis (PCA). Flux distributions were randomly sampled 10,000 times for both the conventional and enzyme-constrained models, and the resulting high-dimensional flux data were projected onto their first two principal components (PCs) to visualize the structure and breadth of the feasible solution spaces. Complementing this, Flux Variability Analysis (FVA) was performed to quantify the range of permissible fluxes for each reaction, with the median flux variability calculated to summarize the overall solution space contraction. We subsequently mapped reactions to metabolic pathways defined by the KEGG database [32]. These subsystems were classified into three categories (Supplementary Information 1) adapted from Bar-Even et al. [33] comprising carbohydrate and energy primary metabolism (CE), amino acid, fatty acid, and nucleotide primary metabolism (AFN), and intermediate and secondary metabolism (ISO). Finally, we calculated the total pathway enzyme usage and the average reduction in flux variability for each defined subsystem.

We next assessed the models' ability to predict physiological phenotypes across diverse nutrient conditions. The growth rates and acetate secretion rates for both ecGEMs and conventional GEMs were simulated on a set of single carbon sources, with substrate uptake rates constrained to experimentally measured values [31]. The model predictions were then quantitatively compared against experimental measurements by calculating Pearson's correlation coefficients, allowing us to evaluate not only absolute accuracy but also the consistency of trends across environmental conditions.

Finally, to test the capacity of the models to replicate complex behavior, such as overflow metabolism, we simulated the response to a gradual increase in glucose availability. The glucose uptake rate was systematically ramped, and at each step, the resulting growth rate, acetate secretion, and oxygen consumption were predicted. The simulated dynamics of overflow metabolism from both the conventional and enzyme-constrained models were compared against well-established experimental characterizations of this phenomenon in *E. coli* [31].

### 2.4 Training dataset

**Fig. 3:**
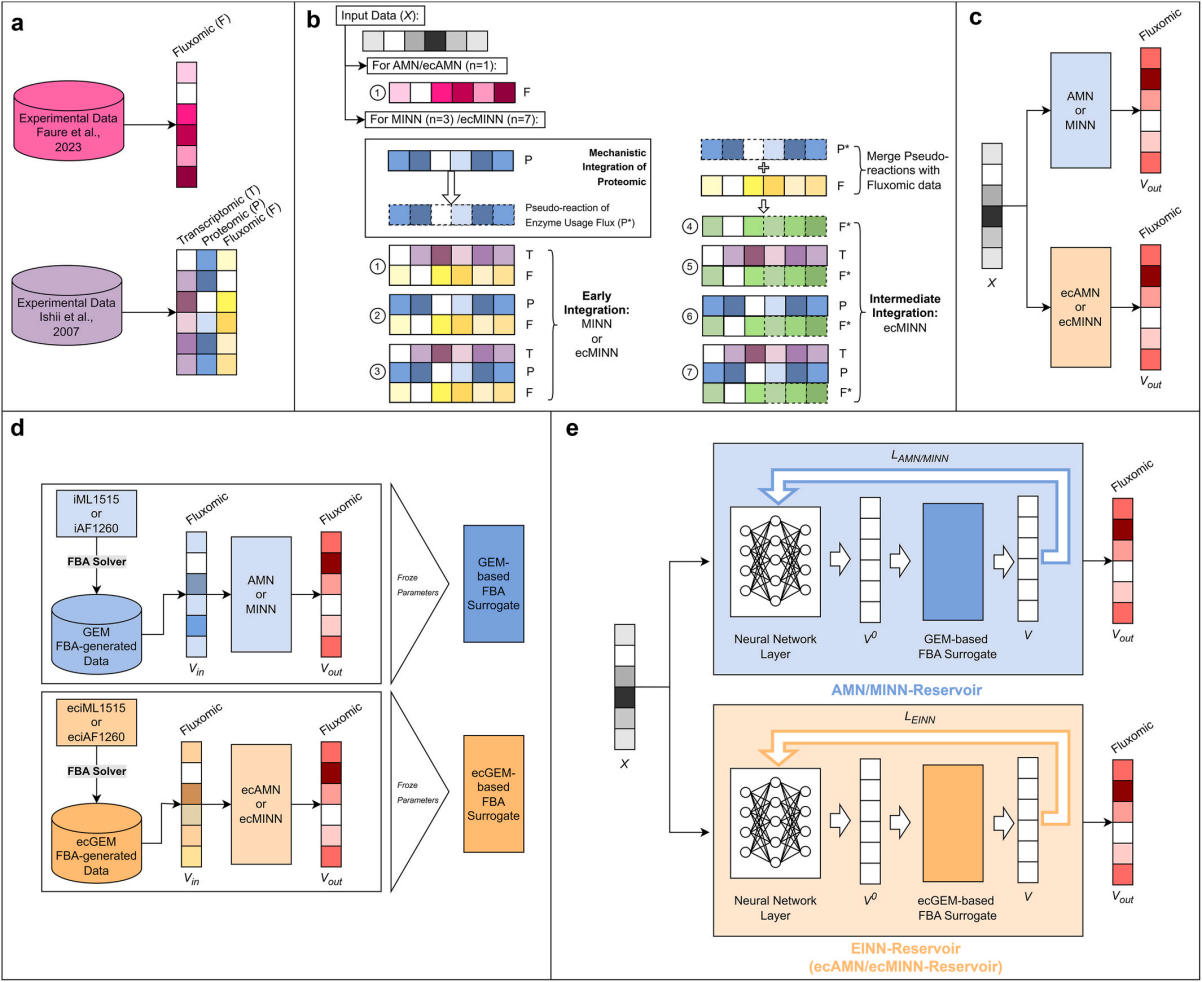
**Schematic overview of the data processing pipeline and training configurations. a** Experimental data sources utilized for model training, including fluxomic data from Faure et al. [14] for the AMN/ecAMN workflow and multi-omics data from Ishii et al. [34] for the MINN/ecMINN workflow. **b** Input feature representation strategies (*X*). The diagram illustrates the varying combinations of fluxomic (F), transcriptomic (T), and proteomic (P) data utilized. For the ecMINN framework, an intermediate integration strategy is employed where proteomic abundances are mechanistically converted into enzyme usage flux pseudo-reactions and merged with fluxomic data (F*). Scenarios 1–3 represent early integration, while 4–7 represent intermediate integration. **c** High-level workflow contrasting the baseline models (AMN/MINN) against the proposed enzyme-constrained variants (ecAMN/ecMINN). **d** Pre-training of reservoir components using synthetic flux data. Synthetic flux distributions are generated via FBA simulations of standard GEMs (iML1515, iAF1260) and their enzyme-constrained counterparts (eciML1515, eciAF1260). These distributions are used to train the hybrid models and establish frozen parameters for the FBA surrogates. **e** Detailed architecture of the reservoir-based models. The upper panel (blue) depicts the baseline AMN/MINN-Reservoir utilizing a standard GEM-based surrogate and minimizing *L*_AMN/MINN_. The lower panel (orange) illustrates the EINN-Reservoir (ecAMN/ecMINN) utilizing an ecGEM-based surrogate and minimizing the composite loss function *L*_EINN_.

For all training of AMN and MINN models in this study, we used the same dataset provided by each respective study (Fig. 3a). To ensure a fair comparison between the original AMN and MINN frameworks and their enzyme-constrained extensions, we used the exact same data-generation protocols established in the original studies [14,21].

#### 2.4.1 Experimental data sources

For the ecAMN framework, we utilized the experimental dataset generated by Faure et al. [14] that was used to train AMN, consisting of 110 growth data of *E. coli* DH5α on unique M9 minimal media compositions supplemented with various carbon sources and amino acids. These conditions comprised mixtures of up to four carbon sources chosen from ribose, maltose, melibiose, trehalose, fructose, galactose, acetate, lactate, succinate, and pyruvate.

For the ecMINN framework, we utilized the multi-omics dataset (ISHII dataset) published by Ishii et al. [34]. This dataset comprises 29 chemostat experiments of *E. coli* K-12 grown in glucose minimal medium, including wild-type strains at five distinct dilution rates (*D* = 0.1 to 0.7 h^−1^) and 24 single-knockout mutant strains at a fixed dilution rate (*D* = 0.2 h^−1^). The dataset provides comprehensive transcriptomic profiles via microarrays, proteomic abundances via LC-MS/MS, and metabolic flux distributions estimated using 13C-Metabolic Flux Analysis (13C-MFA). It is important to note that the original fluxomics data from Ishii et al. [34]. were derived using a core metabolic model. Consequently, these fluxes lie outside the feasible solution space of genome-scale models due to the biomass precursor requirements of the full-scale network. To resolve this mechanistic conflict, following previous research [6,21], we applied a Euclidean projection method (Supplementary Information 2). Each experimental flux vector was projected onto the closest feasible flux distribution within the eciAF1260 solution space under the appropriate dilution rate constraint. This step ensures stoichiometric consistency and removes infeasible combinations of fluxes that would otherwise prevent hybrid model training, effectively reconciling the experimental measurements with the detailed constraints of the genome-scale reconstruction. Importantly, this projected flux data was utilized exclusively for training the ecMINN framework. The baseline MINN models were trained using the unmodified dataset to maintain consistency with the original methodology detailed in the beginning of Section 2.4.

#### 2.4.2 In silico FBA-based synthetic data generation

To generate the necessary synthetic training data, we performed FBA simulations (Fig. 3d) using the ecGEMs of the respective genome-scale models to account for proteomic constraints with maximizing growth rate as the objective function. For the ecAMN model, synthetic flux distributions were generated using the eciML1515. We defined a set of seven obligate uptake reactions necessary for growth (CO_2_, H^+^, H_2_O, NH_4_, O_2_, phosphate, and glycerol) alongside essential salts, ions, and four amino acids (alanine, proline, threonine, and glycine) to mirror the experimental conditions. The upper bounds for these obligate reactions were set to 10 mmol gDW^−1^h^−1^. To simulate varying media compositions, variable uptake reactions were selected from the experimental training set using a binomial distribution. The upper bounds for these variable reactions were drawn from a uniform distribution between 0 and 5.3 mmol gDW^−1^h^−1^, a threshold chosen to align predicted growth rates with experimentally observed ranges [14].

For the ecMINN architecture, we generated a synthetic dataset to train the reservoir component by running 10,000 FBA simulations using the eciAF1260 to approximate linear programming behavior under varying environmental conditions. Uptake and secretion rates for key exchange metabolites, specifically glucose, oxygen, carbon dioxide, ethanol, and acetate, were randomly sampled within the range of variability observed in the Ishii et al. [34] dataset.

### 2.5 Training configurations

To ensure a rigorous benchmark, the original AMN, MINN, AMN-Reservoir, and MINN-Reservoir models were re-implemented exactly as described in their respective publications [14,21]. For ecAMN and the AMN baseline, the input layer receives the medium composition vector with the scalar growth rate serving as the evaluation target. For ecMINN and the MINN baseline, inputs were structured to test multi-omics integration. This included the fluxomic input (F) of glucose and oxygen fluxes, augmented with transcriptomic (T) and proteomic (P) features in various combinations (Fig. 3b). The ecMINN architecture exclusively utilized an intermediate strategy (F*) comprising the full projected flux vector supplemented with proteome-derived enzyme capacity constraints. The output layer dimension for all models corresponds to the number of reactions in the respective unidirectional (ec)GEM, providing the initial flux estimate ***V***^0^.

Training protocols were categorized into standard training (Fig. 3c) and two-stage training procedure (Fig. 3d and e). All models utilized the Adam optimizer, but loss functions varied by architecture where EINN variants minimized the composite loss *L*_EINN_ (Equation (4)), while baseline models utilized their original loss formulations from Faure et al. [14] and Tazza et al. [21]. Standard models (ecAMN, ecMINN, AMN, MINN) were trained directly on experimental datasets. For models utilizing the reservoir approach (ecAMN-Reservoir, ecMINN-Reservoir, and their baseline equivalents), the base model was pre-trained as a surrogate FBA solver on synthetic data generated by simulations with randomized uptake bounds. Once the “reservoir” weights were frozen, a new neural layer was attached and trained on experimental data to predict optimal constraints. The specific hyperparameters for EINN models including learning rates, batch sizes, and epoch limits are detailed in Table 4.

Crucially, the utilization of mathematically optimal FBA data (where the objective function is maximized) during the pre-training phase is a deliberate architectural requirement. This phase teaches the network the absolute physical boundaries of the mass-balanced and thermodynamically feasible solution space. Freezing these weights establishes a strictly compliant mechanistic surrogate. To bridge the distribution shift between these optimal pre-training states and sub-optimal biological reality, the unfrozen upstream neural layer acts as a dynamic regulatory mechanism. By learning to restrict the predicted input bounds ***V***_in_ based on experimental data, the upstream layer effectively restrains the optimal surrogate, forcing it to output the observed, non-maximized physiological states.

### 2.6 Cross-validation and evaluation metrics

Models utilizing the growth phenotype dataset (AMN and ecAMN) employed a repeated 5-fold cross-validation scheme across 200 seeds. To rigorously evaluate model generalization under unseen environmental scenarios, these models were additionally evaluated using a Leave-One-Substrate-Out (LOSO) cross-validation framework. Conversely, models utilizing the multi-omics ISHII dataset (MINN and ecMINN) were evaluated using an outer leave-one-out cross-validation (LOOCV) loop with repetition across 10 seeds. To specifically assess their ability to generalize flux predictions to unseen genetic perturbations, this procedure functioned as a Leave-One-Mutant-Out (LOMO) cross-validation strategy.

Model performance was evaluated using task-specific metrics consistent with prior studies. For the AMN and ecAMN frameworks, which perform growth rate regression, we report the cross-validated coefficient of determination (Q^2^) alongside the validation loss value. For the MINN and ecMINN frameworks, which perform multivariate flux prediction, we report the regression coefficient (R^2^), Mean Absolute Error (MAE), Root Mean Squared Error (RMSE), and Normalized Error (NE) to assess prediction accuracy across scales, supplemented by the stoichiometric (***SV***) loss to evaluate constraint violations. All metrics are presented as the mean ± standard deviation across cross-validation folds.

### 2.7 Evaluation of carbon source utilization strategy

**Fig. 4:**
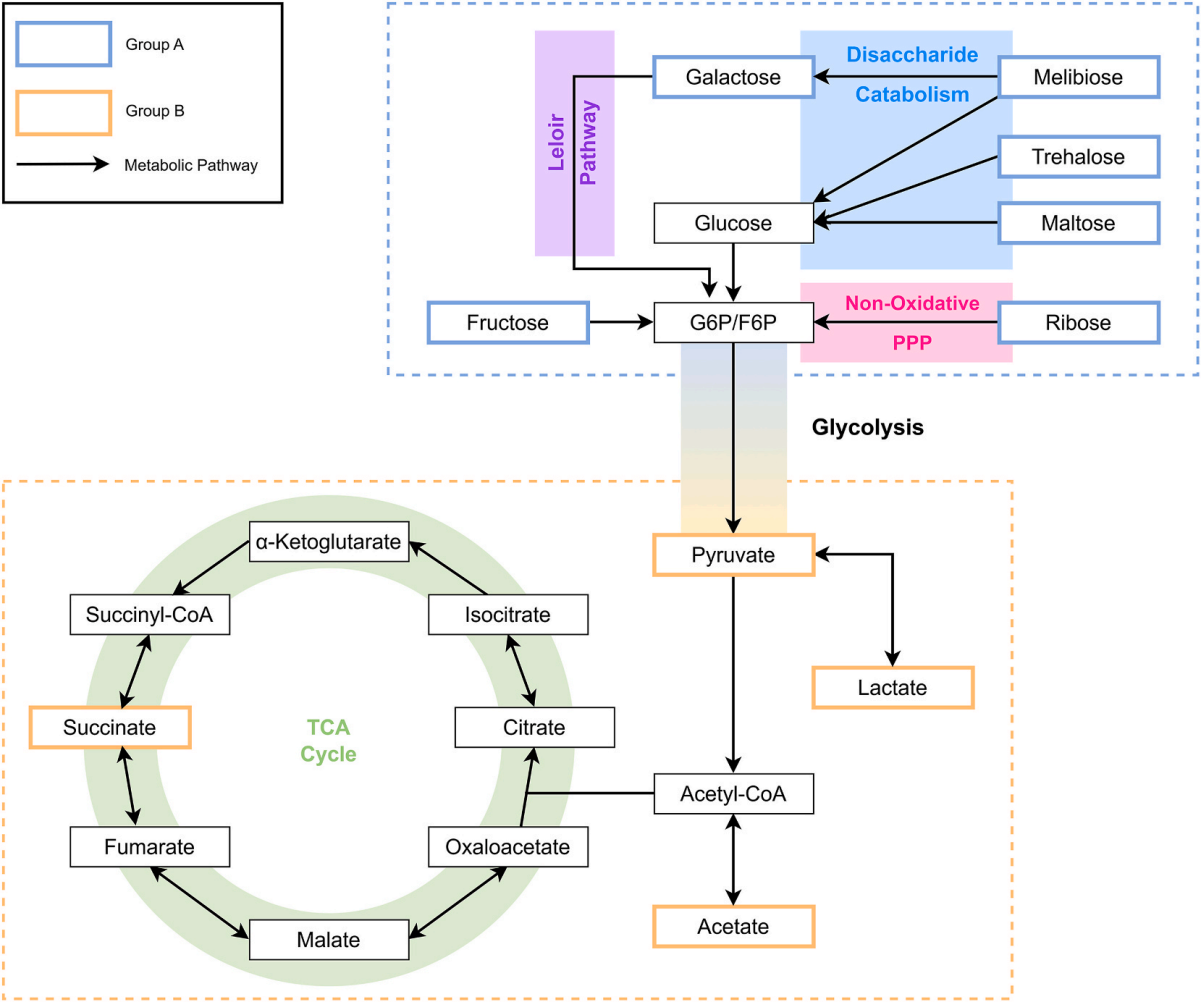
**Metabolic topology of carbon source classification.** Substrates are partitioned into Group A (blue) and Group B (orange) based on their entry points relative to central carbon metabolism. Group A substrates, including sugars such as galactose and maltose, converge at the glucose-6-phosphate/fructose-6-phosphate (G6P/F6P) node via peripheral pathways (e.g., Leloir pathway, non-oxidative PPP) prior to upper glycolysis. In contrast, Group B substrates enter metabolism downstream at the level of pyruvate, acetyl-CoA, or directly into the tricarboxylic acid (TCA) cycle intermediates.

To assess the biological realism of the AMN-reservoir and ecAMN-reservoir models, we evaluate how each model can predict the expected phenotype for carbon source utilization according to the specific composition of the experimental medium and the classification established by Wang et al. [35], despite these rules not being part of the training objective. In this framework, substrates are differentiated by their metabolic entry points. Carbon sources from Group A converge to the G6P/F6P node before entering various pools, while those from Group B can take alternative routes (Fig. 4). This Group A/B partitioning and the branch-efficiency rationale motivate why mixtures of A + A typically generate diauxic behaviour while A + B mixtures often permit co-utilization [35].

For each data point from experimental data [14], the expected phenotype was derived by applying empirical topological rules of the metabolic network to the available substrates. Conditions in which the medium contained multiple Group A substrates with no Group B substrate (pure multiauxie) were excluded from the rule-based evaluation because such dynamics are intrinsically time-dependent and therefore outside the representational scope of steady-state FBA or any FBA-based reservoir model. For the remaining valid conditions, the criteria for correctness were:

1. Single Source: The model must utilize the specific available substrate (exact match).
2. Co-utilization: In mixed environments, correctness requires either the hierarchical consumption of exactly one Group A source alongside Group B sources, or the simultaneous consumption of multiple Group B sources (*n *≥ 2) in the absence of Group A.

To quantitatively evaluate the model's performance against these expectations, “accuracy” was defined as a binary classification metric representing the percentage of valid conditions where the predicted uptake behavior strictly adhered to the aforementioned rules. It is computed as follows:

(5) $$\text{Accuracy}\left(\%\right)=\frac{N_{\mathrm{compliant}}}{N_{\mathrm{valid}}}\times 100$$

where *N*_compliant_ represents the number of experimental conditions where the model's predicted carbon substrate uptake perfectly matches the rule-based criteria, and *N*_valid_ represents the total number of valid experimental conditions evaluated (excluding pure multiauxie conditions).

### 2.8 Computational workflow implementation

We operationalized the EINN framework within a modular Nextflow workflow to bridge MATLAB-based ecModel construction and Python-based ecModel preprocessing. Enzyme-constrained model reconstruction was performed in MATLAB (R2023b) using the GECKO Toolbox 3.0. The resulting ecGEMs were exported to a Python environment (Python 3.10), where synthetic training data were generated using CobraPy (v0.26.3) with the Gurobi optimizer (v12.0.1, academic license). Subsequently, a dedicated TensorFlow/Keras environment (TensorFlow 2.13, Keras 2.13) was initialized to train the hybrid neural-mechanistic architectures, optimizing the composite loss function against the generated datasets.

## 3 Results

### 3.1 Construction of enzyme-constrained iML1515 and iAF1260 using GECKO 3.0

To power these hybrid models with the state-of-the-art enzyme kinetic constraining method, we constructed new *E. coli* ecGEMs using the latest GECKO 3.0 toolbox [26]. While GECKO has been successfully applied to *E. coli* model in earlier version [9], GECKO 3.0 introduced important refinements to the proteome-allocation framework, including a unified enzyme-cost formulation and expanded *k*_cat_ coverage through deep-learning predictions. Thus, a systematic validation of *E. coli* ecGEMs constructed under this updated formulation has not yet been performed. To ensure direct and fair comparisons when benchmarking our ecAMN and ecMINN against their established counterparts, we built ecGEMs for the specific base models each original framework uses (eciML1515 for AMN and eciAF1260 for MINN). This approach guarantees that any performance improvements are attributable to the incorporation of enzyme constraints rather than to differences in the underlying metabolic network.

Starting from the base models used in the AMN (iML1515) and MINN (iAF1260) frameworks, we integrated enzyme turnover data and proteomic allocation constraints to generate eciML1515 and eciAF1260, respectively. This process expanded the models to include explicit pseudo-reactions for enzyme usage, resulting in the specifications detailed in Table 1.

**Table 1:** Specifications of Genome-Scale Metabolic Models and their Enzyme-Constrained Counterparts.

Property	iML1515	eciML1515	iAF1260	eciAF1260
Total Reactions	2712	7409	2382	6234
Original reactions	2712	5895	2382	4983
Pseudo-reactions (Enzyme Usage)	n/a	1514	n/a	1260
Total Metabolites	1877	3392	1668	2929
Original Metabolites	1877	1877	1668	1668
Pseudo-metabolites (Enzyme)	n/a	1515	n/a	1261

**Fig. 5:**
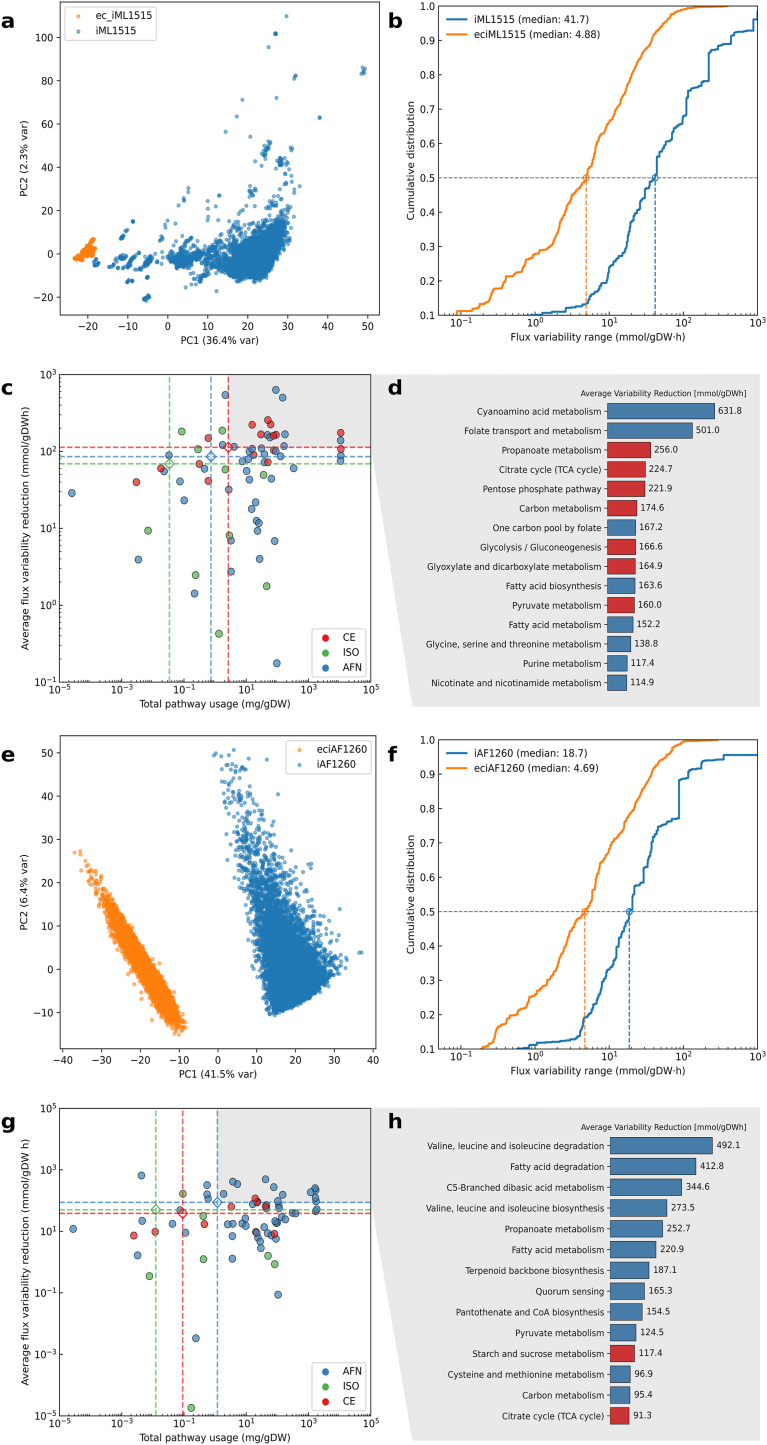
**Comparison of flux variability and enzyme usage across *E. coli* models. a–d** Comparison between the stoichiometric model iML1515 and its enzyme-constrained counterpart eciML1515; **e–h** corresponding analysis for iAF1260 and eciAF1260. **a, e** PCA of 10,000 random flux samples shows that imposing enzyme capacity constraints produces distinct clustering and reduces the dispersion of flux states. **b, f** Cumulative distributions of flux variability ranges highlight a substantial contraction of the feasible solution space in the enzyme-constrained models. **c, g** Relationship between average flux variability reduction and total pathway enzyme usage. Pathways are categorized into three groups: Carbohydrate and energy primary metabolism (CE; red markers); Amino acid, fatty acid, and nucleotide primary metabolism (AFN; blue markers); and Intermediate and secondary metabolism (ISO; green markers). The dashed lines indicate the mean values for total pathway usage (vertical) and variability reduction (horizontal). The data demonstrates that pathways with higher enzyme investment generally experience greater restrictions in flux flexibility. **d, h** Pathways exhibiting the largest average reductions in flux variability.

To establish the suitability of these ecGEMs for hybrid modeling, we first evaluated how enzyme constraints alter the predicted landscape of metabolic fluxes. Comparing ecGEMs to their conventional counterparts revealed that introducing enzyme capacity constraints led to markedly different flux distributions. PCA of flux samples revealed that conventional models exhibited broad, diffuse distributions with significant variance along the PCs (Fig. 5a–e), reflecting a large and flexible solution space. In distinct contrast, the ecGEMs consistently formed compact, tight clusters. This demonstrates that enzyme constraints significantly prune the feasible flux landscape, effectively collapsing the broad phenotypic potential of the conventional models into a more defined and biologically constrained state. Flux variability analysis (FVA) further demonstrated the restrictive effect of enzyme constraints on the feasible solution space where the median variability dropped from 41.7 to 4.88 mmol gDW^−1^ h^−1^ for iML1515-eciML1515 and from 18.7 to 4.69 mmol gDW^−1^ h^−1^ for iAF1260-eciAF1260 (Fig. 5b–f). These distributions showed an order of magnitude compression of allowable flux ranges in the ecGEMs relative to their unconstrained counterparts. Pathway-level analyses revealed that reactions belonging to pathways with high total enzyme usage (e.g., central carbon and amino acid metabolism) experienced the largest reductions in variability (Fig. 5c–g). Aggregated pathway summaries further indicated that the strongest constraint effects occurred in cyanamino acid metabolism, folate metabolism, and the pentose phosphate pathway in eciML1515, whereas eciAF1260 showed the largest reductions in valine/leucine/isoleucine degradation, fatty acid degradation, and branched-chain metabolism (Fig. 5d–h).

**Fig. 6:**
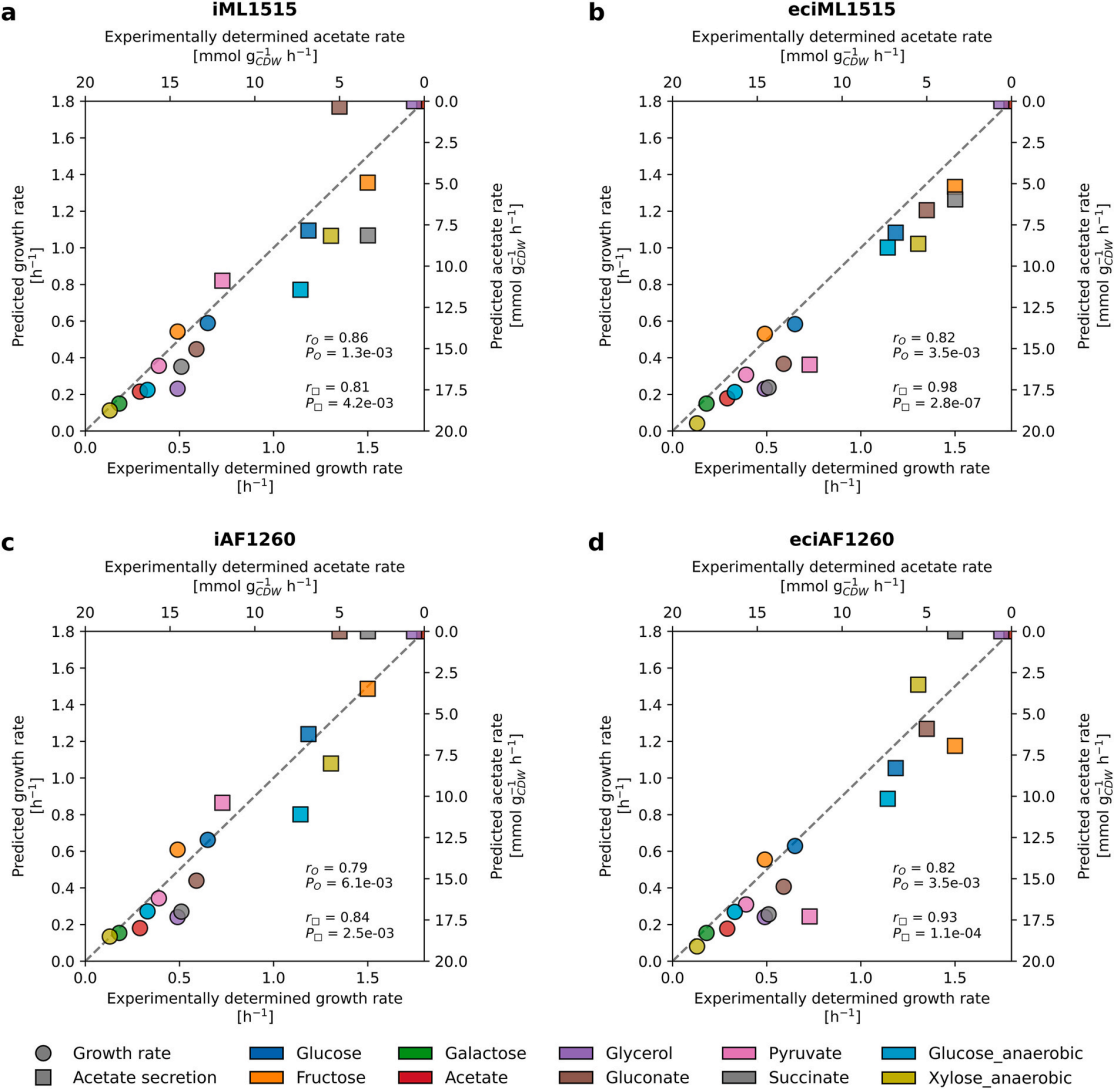
**Comparison of predicted and experimentally measured growth and acetate secretion rates across multiple carbon sources for conventional and enzyme-constrained models.** Scatter plots compare model-predicted growth rates and acetate secretion rates with experimental measurements across diverse carbon substrates for **a** iML1515, **b** eciML1515, **c** iAF1260, and **d** eciAF1260. Each point corresponds to a distinct carbon source, with colors indicating substrates and marker shapes distinguishing growth rates (circles) from acetate secretion rates (squares).

Building on this constrained solution space, we next investigated whether the ecGEMs could translate this into improved biological realism across diverse nutrient environments. When comparing simulated and experimentally measured rates (Fig. 6), both ecGEMs and GEMs maintained similar high accuracy in predicting growth rate (*r* ≈ 0.8 across all models), indicating that the addition of enzyme constraints does not compromise basic growth rate predictions. However, the enzyme-constrained models demonstrated a marked improvement in predicting metabolic by-products. The ecGEMs prediction yielded higher correlations for acetate secretion, capturing condition-specific physiological responses that unconstrained models often miss.

**Fig. 7:**
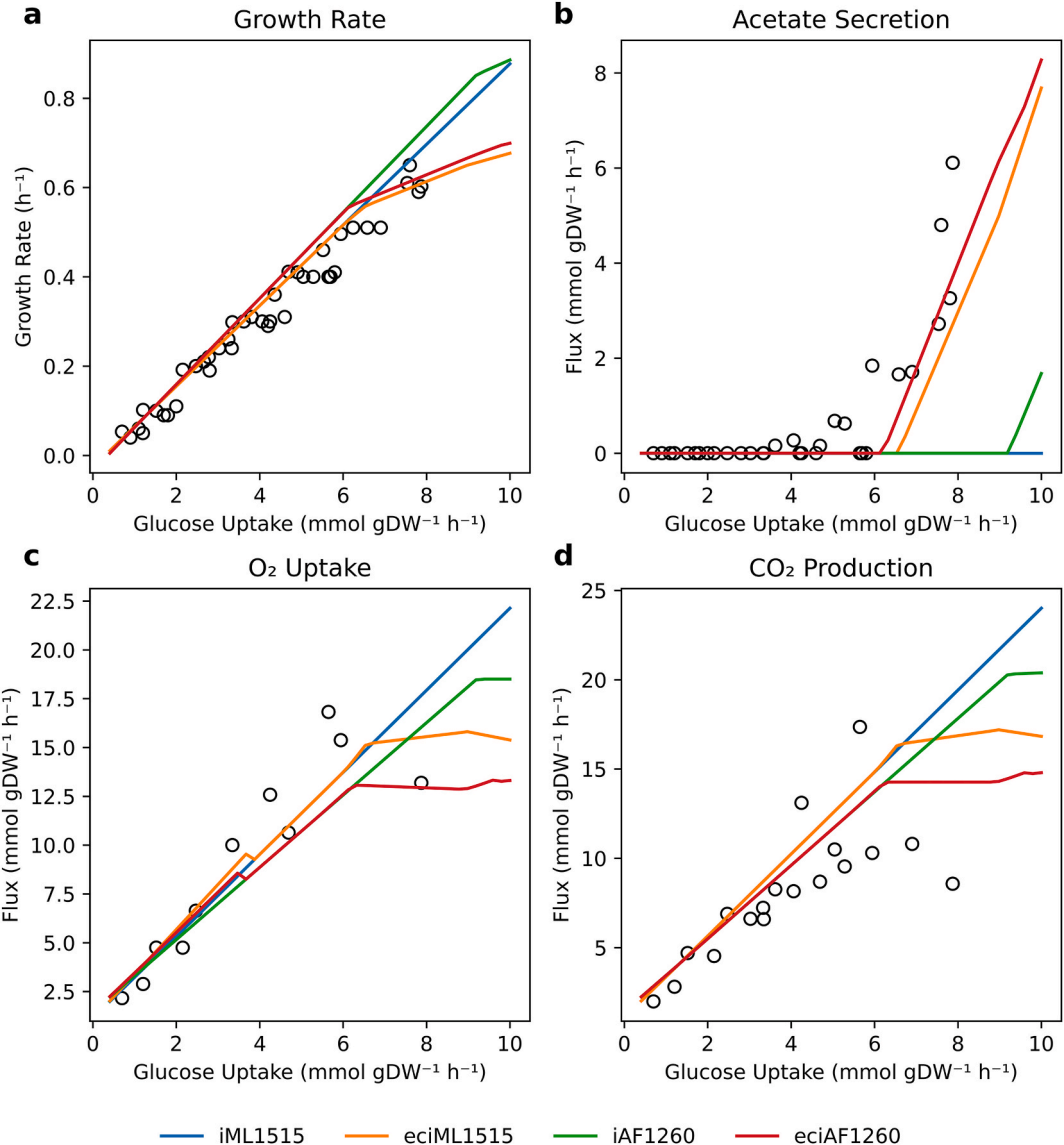
**Comparison of predicted metabolic phenotypes from GEMs and their enzyme-constrained counterparts across increasing glucose uptake rates.** Four key fluxes were evaluated: **a** growth rate, **b** acetate secretion, **c** O_2_ uptake, and **d** CO_2_ production. Experimental measurements are overlaid as open circles. This analysis is used to validate whether the constructed ecGEMs behave correctly, particularly in reproducing physiologically relevant phenomena such as proteome-limited growth and acetate overflow.

Finally, to assess their ability to replicate complex emergent behaviors, we tested whether the ecGEMs could capture overflow metabolism (Fig. 7), which is a key physiological phenomenon that conventional GEMs typically fail to reproduce [36]. When simulated across increasing glucose uptake rates, conventional GEMs exhibited linear scaling of growth, acetate secretion, and respiratory fluxes that exceeded known physiological bounds. In contrast, the ecGEMs displayed qualitatively consistent proteome-limited behavior, including growth rate saturation and acetate overflow onset at high glucose uptake, closely matching experimentally observed *E. coli* physiology [31,[37], [38], [39], [40], [41], [42], [43], [44]]. This critical shift from linear to enzyme-limited dynamics demonstrates that ecGEMs intrinsically capture the metabolic trade-offs that give rise to overflow metabolism, a capability lacking in standard GEMs.

In summary, we have shown that ecGEMs fundamentally reshape the metabolic solution space, yielding stable and biologically grounded flux predictions, improve prediction accuracy across diverse environments, and successfully recapitulate complex physiological phenomena like overflow metabolism. These attributes validate the quality of the constructed ecGEMs and establish their suitability for integration with machine learning. Consequently, enzyme-constrained models provide a more physiologically realistic and robust foundation for hybrid modeling than standard GEMs.

### 3.2 Enzyme constraints improve hybrid model accuracy and convergence stability

**Fig. 8:**
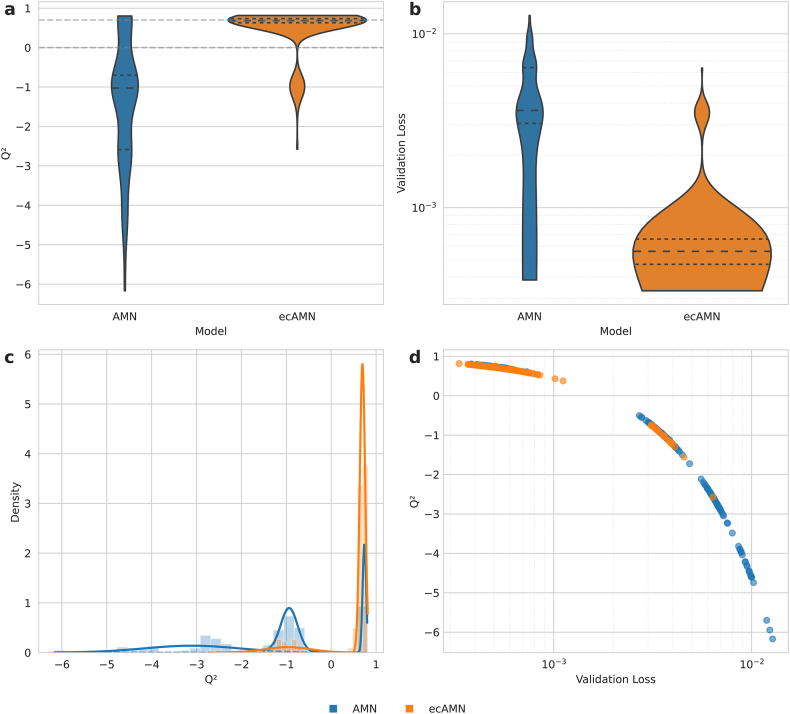
**Comparative performance of AMN and ecAMN models across 200 random seeds. a** Distribution of predictive accuracy (Q^2^) for AMN and ecAMN models. ecAMN displays a markedly tighter and higher-performing Q^2^ distribution whereas AMN exhibits broad dispersion including frequent large negative Q^2^ values. Dashed lines indicate Q^2^ = 0 and Q^2^ = 0.7 performance thresholds. **b** Validation loss distributions on a log scale. ecAMN achieves substantially lower and more consistent losses compared to AMN, indicating improved generalization. **c** GMM-based density estimation of Q^2^ distributions. The ecAMN mixture is dominated by a high-Q^2^ mode, whereas AMN shows multi-modal behavior reflecting unstable performance. **d** Joint scatter of Q^2^ and validation loss across all runs. ecAMN simulations form a compact, high-performance cluster characterized by simultaneously low validation loss and high Q^2^, whereas AMN runs are widely dispersed across the joint space, reflecting heterogeneous performance and entrapment in multiple suboptimal basins.

To evaluate the impact of incorporating enzyme capacity constraints into the AMN framework, we trained both AMN and ecAMN across 200 random initializations (Fig. 8, Table 2). Visual inspection of the performance distributions (Fig. 8a and b) reveals a contrast in convergence consistency, whereas AMN exhibited a sparse, vertically elongated distribution characterized by high variance, ecAMN results were densely concentrated in regions of high predictive accuracy (Q^2^) and low validation loss. Consequently, ecAMN substantially outperformed AMN in the vast majority of runs, revealing highly significant improvements for ecAMN (Mann–Whitney *U* test; *p* = 2.5 × 10^−25^ for Q^2^; *p* = 2.3 × 10^−28^ for validation loss) with large effect sizes (Cliff's delta = 0.60 and 0.64, respectively).

**Table 2:** Predictive performance and validation loss of AMN and ecAMN models.

Model	Mean Q^2^	Median Q^2^	Std Q^2^	Min Q^2^	Max Q^2^	Q^2^ > 0.7%	Q^2^ ≤ 0%	Mean Val Loss	Median Val Loss	Std. Val Loss
AMN	−1.3771	−1.0245	1.6111	−6.1684	0.8025	18.5	78.5	0.0043	0.0036	0.0029
ecAMN	0.4640	0.6872	0.6122	−2.5748	0.8150	43.0	13.0	0.0010	0.0006	0.0011

The proportion of non-predictive models (Q^2^ ≤ 0) decreased from 78.5% in AMN to 13% in ecAMN, whereas the fraction of high-performing runs (Q^2^ > 0.7) increased from 18.5% to 43% (Table 2). Gaussian mixture modeling of Q^2^ distributions showed that ecAMN converged predominantly to a single high-performance mode (mean Q^2^ = 0.693, weight 0.87), with only minor low-performance components (Fig. 8c). By contrast, AMN exhibited a trimodal distribution comprising high-performance (mean Q^2^ = 0.74, weight 0.22), moderate-performance (mean Q^2^ = −0.94, weight 0.42), and poor-performance (mean Q^2^ = −3.1, weight 0.37) modes (Fig. 8c). Joint analysis of Q^2^ and validation loss demonstrated that ecAMN runs formed a compact high-performance cluster, whereas AMN runs were broadly scattered, reflecting entrapment in multiple suboptimal basins (Fig. 8d). Unsupervised multivariate clustering in the joint Q^2^–loss space confirmed this separation, where ecAMN yielded a dominant, well-defined cluster (*n* = 174, silhouette score 0.941), compared with a more heterogeneous landscape for AMN (silhouette score 0.672).

Translating this clustered performance landscape into practical deployment reliability, the empirical probability that a randomly initialized ecAMN model will outperform its standard AMN counterpart is 0.801 for Q^2^ and 0.819 for validation loss. Bootstrap resampling (10,000 iterations) confirmed near-certain superiority of ecAMN population means (*P* ≈ 1), with 95% confidence intervals for the mean differences of 1.60–2.08 for Q^2^ and 0.0029–0.0037 for validation loss. Ultimately, these metrics demonstrate that incorporating enzyme capacity constraints largely eliminates the risk of convergence to poor local minima, securing the consistent training stability and predictive performance required for robust usage.

**Fig. 9:**
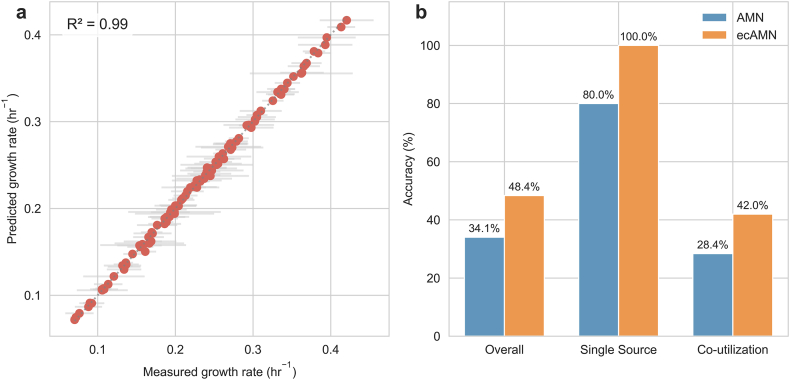
**Performance of ecAMN-reservoir versus AMN-reservoir. a** Measured versus predicted growth rates for the ecAMN-reservoir model. Each point represents one growth condition; horizontal bars denote the standard deviation of the measured growth rate. **b** Evaluation of carbon-source utilization patterns predicted by AMN-reservoirs and ecAMN-reservoirs using biologically motivated rules derived from established substrate-uptake behavior in *E. coli*. Accuracy is shown for overall cases, single-source conditions, and co-utilization scenarios involving combinations of Group A and Group B substrates. Here, accuracy represents the percentage of valid experimental conditions where the predicted carbon uptake perfectly matched the expected biological rules (detailed in Section 2.7).

We then benchmarked the ecAMN-reservoir against experimentally measured growth rates. Using the same test set as for the AMN-reservoir, the ecAMN-reservoir attained R^2^ = 0.99 (Fig. 9a), compared with the previously reported R^2^ = 0.97 for the AMN-reservoir [14], indicating improved performance in steady-state growth prediction. To determine if this quantitative gain translated to more biologically plausible substrate uptake, we evaluated predictions against established rules of carbon utilization (Fig. 9b), excluding cases involving only more than one Group A sugars as their time-dependent, hierarchical uptake cannot be captured by steady-state modeling. Among the remaining cases (*n* = 80), we distinguished single-source conditions (exactly one active carbon source) and co-utilization cases (multiple available substrates). For single-source conditions the ecAMN-reservoir reached 100% agreement with the mechanistic expectation (10/10), outperforming the AMN-reservoir (8/10). In co-utilization cases (*n* = 70) both models showed substantially lower absolute agreement with the rules. This is a predictable outcome because steady-state models lack the temporal resolution to correctly handle multiple preferred carbon sources. When more than one Group A source is available, steady-state simulations often predict simultaneous uptake to maximize growth even though the organism consumes them sequentially. Thus, while absolute co-utilization accuracy remains limited by the nature of steady-state assumption of GEMs, ecAMN-reservoir improved co-utilization accuracy from 28.4% to 42.0%.

Overall, accuracy across all non-excluded cases rose significantly (McNemar test, *p =* 2.44 × 10^−4^) from 34.1% (AMN-reservoir) to 48.4% (ecAMN-reservoir), with many instances where ecAMN-reservoir corrected AMN-reservoir's failures. These results indicate that adding enzyme-constraint information into the reservoir provides a more informative mechanistic prior that improves both growth-rate prediction and the consistency of inferred uptake patterns under steady-state assumptions. To identify the specific conditions driving this improvement, we breakdown the individual predictions (Supplementary Information 3) and we observed that the standard AMN-reservoir consistently failed to predict phenotypes involving the uptake of Group B carbon sources (organic acids and TCA intermediates). Specifically, standard AMN-reservoirs dropped Group B carbon source signals entirely in single-source medium and failed to predict carbon source co-utilization when Group A carbon source was present in the medium. The ecAMN-reservoir successfully resolved these specific blind spots, accounting for the vast majority of the corrected predictions.

### 3.3 Mechanistic integration of proteomics optimizes the performance trade-off in enzyme-constrained multi-omics models

**Fig. 10:**
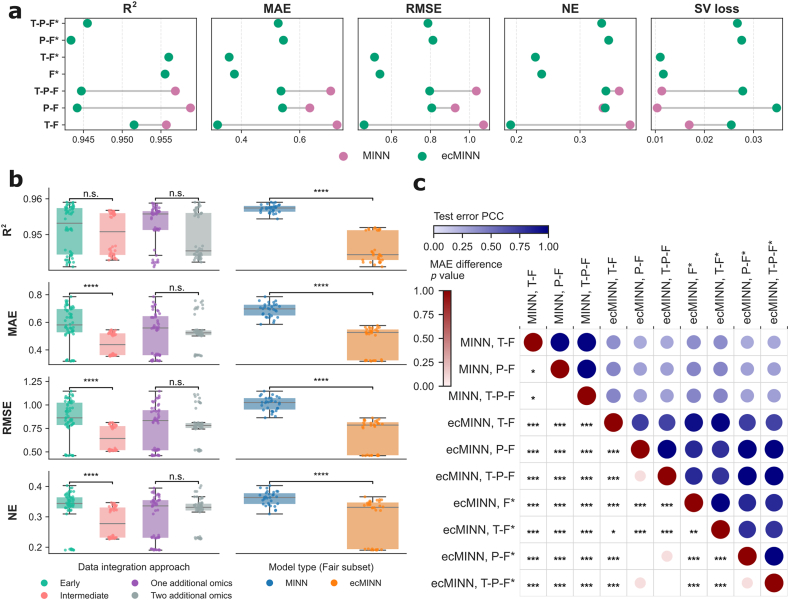
**Comprehensive performance evaluation and statistical characterization of MINN and ecMINN architectures. a** Performance trade-off analysis. A dumbbell plot contrasts the predictive capabilities of unconstrained MINN (orange) and enzyme-constrained ecMINN (blue) models across five quantitative metrics. The connecting lines visualize the systematic shift in the solution space: ecMINN variants consistently minimize absolute and normalized errors (MAE, RMSE, NE) and structural variance (***SV*** loss), while MINN variants retain marginally higher global explained variance (R^2^). **b** Statistical impact of data integration strategies. Box plots display the distribution of performance. “Early” integration (green) treats omics as input features, while “Intermediate” integration (pink) embeds proteomic data as mechanistic constraints (F*). Statistical significance was assessed using the Mann-Whitney *U* test (*: *p* < 0.01, **: *p* < 0.001, ***: *p* < 0.0001, n.s.: not significant). The comparison between “One additional omics” and “Two additional omics” illustrates the diminishing returns and potential “over-noising” effect of increasing data complexity. **c** Architectural divergence and feature redundancy. A symmetric matrix comparing pairwise model behaviors. The upper triangle shows the Pearson Correlation Coefficient (PCC) of test errors; dark blue indicates high intra-model consistency, while the light blue off-diagonal region reveals weak correlation between MINN and ecMINN. The lower triangle reports the statistical significance of MAE differences (Mann-Whitney *U* test).

We conducted a comprehensive performance evaluation of the MINN and ecMINN architectures using leave-one-out cross-validation (LOOCV). The fundamental trade-off between the two model families is evident (Fig. 10a). A distinct separation exists where unconstrained MINN models generally achieve marginally higher explained variance (R^2^) but suffer from substantially higher error rates (MAE, RMSE, NE). Conversely, enzyme-constrained ecMINN models demonstrate a dramatic reduction in error metrics, shifting the performance distribution toward higher precision.

Among all evaluated models (Table 3), the mechanistic integration of proteomics strategy with additional transcriptomic data (ecMINN-T-F*) achieved the lowest error metrics across the board. While other configurations may excel in a single metric, ecMINN-T-F* consistently ranks among the top performers across the entire spectrum of evaluation criteria. Notably, it achieves a low ***SV*** loss comparable to the stable unconstrained models, and maintains a high explained variance that rivals the MINN baseline. This balance suggests that ecMINN-T-F* successfully avoids the overfitting often associated with high R^2^, while also preventing the high ***SV*** loss seen in other constrained variants. However, mechanistic integration of proteomics without additional transcriptomic data (ecMINN-F*) achieved performance nearly identical to this top-tier configuration. Given this minimal magnitude of improvement provided by the added complexity, ecMINN-F* may emerge as the most efficient configuration. This suggests that while adding transcriptomics (T) offers a slight numerical edge, the core mechanistic constraints in F* are sufficient to capture the essential biological signal.

**Table 3:** Performance evaluation of MINN and ecMINN models.

Model	Data	R^2^	MAE	RMSE	NE	*SV* loss
MINN	T-F	0.9561 ± 0.0582	0.7206 ± 1.0476	1.0583 ± 1.5133	0.3728 ± 0.5303	0.0162 ± 0.0242
	P–F	0.9579 ± 0.0578	0.6308 ± 0.7743	0.9250 ± 1.0718	0.3357 ± 0.3841	0.0108 ± 0.0068
	T-P-F	0.9578 ± 0.0576	0.7154 ± 0.9485	1.0492 ± 1.3546	0.3727 ± 0.4706	0.0139 ± 0.0194
ecMINN	T-F	0.9515 ± 0.0713	0.3231 ± 0.3793	0.4629 ± 0.5446	0.1921 ± 0.1415	0.0241 ± 0.0478
	P–F	0.9427 ± 0.0681	0.5580 ± 0.5052	0.8323 ± 0.7806	0.3518 ± 0.1861	0.0357 ± 0.0358
	T-P-F	0.9440 ± 0.0688	0.5251 ± 0.5052	0.7829 ± 0.7771	0.3319 ± 0.2025	0.0282 ± 0.0291
	F*	0.9560 ± 0.0619	0.3654 ± 0.3489	0.5224 ± 0.4917	0.2331 ± 0.1705	0.0119 ± 0.0132
	T-F*	0.9559 ± 0.0630	0.3609 ± 0.3562	0.5179 ± 0.5033	0.2300 ± 0.1722	0.0108 ± 0.0113
	P–F*	0.9438 ± 0.0675	0.5335 ± 0.4936	0.7940 ± 0.7556	0.3366 ± 0.1921	0.0305 ± 0.0305
	T-P-F*	0.9452 ± 0.0683	0.5141 ± 0.4942	0.7656 ± 0.7583	0.3261 ± 0.2076	0.0261 ± 0.0272

T (Transcriptomic), P (Proteomic), F (Fluxomic), F* (Proteomic data mechanistically integrated via pseudo-reaction combined with fluxomic data).

**Table 4:** Training hyperparameters for EINN models.

Model	Learning Rate	Batch Size	Epochs
ecAMN	0.001	5	20
ecMINN	0.00001	5	300
ecAMN-Reservoir (Mechanistic Surrogate Learning)	0.001	100	20
ecAMN-Reservoir (Downstream Parameter Learning)	0.0001	5	1000
ecMINN-Reservoir (Mechanistic Surrogate Learning)	0.00001	5	1000
ecMINN-Reservoir (Downstream Parameter Learning)	0.00001	5	300

We also analyzed the error distributions across integration approaches (Fig. 10b). The analysis confirms the critical value of mechanistic constraints where the Intermediate strategy yielded highly significant reductions in error compared to the Early integration, specifically for MAE (Mann-Whitney *U* test, *p* = 7.70 × 10^−7^), RMSE (Mann-Whitney *U* test, *p* = 7.16 × 10^−7^), and NE (Mann-Whitney *U* test, *p* = 1.80 × 10^−5^). Importantly, this gain in precision did not come at the cost of explanatory power or model stability, as the differences in R^2^ (Mann-Whitney *U* test, *p* = 0.129) and ***SV*** loss (Mann-Whitney *U* test, *p* = 0.250) were statistically insignificant. This confirms that embedding proteomics as mechanistic constraints (F*) refines solution precision without compromising the model's global variance explanation.

The analysis also elucidates the diminishing returns of increasing data complexity (Fig. 10b). Comparing models with one additional omics layer versus two revealed no statistically significant performance gains across any metric (MAE: Mann-Whitney *U* test, *p* = 0.612; RMSE: Mann-Whitney *U* test, *p* = 0.667; NE: Mann-Whitney *U* test, *p* = 0.817; ***SV*** loss: Mann-Whitney *U* test, *p* = 0.176). Even the explained variance showed no significant improvement (R^2^: Mann-Whitney *U* test, *p* = 0.054). This lack of statistical differentiation indicates that the addition of high-dimensional layers like transcriptomics becomes redundant once the core mechanistic constraints are established via F*, as it increases complexity without significantly improving model performance.

To understand the systematic prediction differences between these architectures, we analyzed the pairwise correlation of the prediction outputs (Fig. 10c, upper triangle). The correlation analysis reveals a distinct separation between the MINN and ecMINN families. While models within the MINN family are highly correlated internally, they show weak correlation with ecMINN variants. This suggests that the two architectures likely converge to different local minima during optimization. The introduction of enzyme constraints appears to force the model into a biologically distinct solution space, systematically altering predictions regardless of the input omics.

This divergence is further supported by the additional statistical significance testing of MAE differences (Fig. 10c, lower triangle). Every pairwise comparison between a MINN variant and an ecMINN variant yielded highly significant differences in predictive error (Mann-Whitney *U* test, *p* = 3.02 × 10^−11^). To explicitly test the hypothesis of data redundancy, given that F* represents proteomics data mechanistically integrated into flux constraints, our experimental design included configurations like ecMINN-P-F* and ecMINN-T-P-F* where proteomics data is used simultaneously as both a raw feature and a mechanistic constraint. The results confirm that this dual usage yields no performance benefit. Specifically, the ecMINN-T-P-F and ecMINN-P-F variants showed no statistically significant difference in MAE when compared to the structurally redundant ecMINN-P-F* and ecMINN-T-P-F* configurations. Consequently, the simpler ecMINN-F* architecture is validated as the most efficient approach because it achieves optimal precision without the computational overhead of processing redundant feature layers.

### 3.4 Enzyme constraints enhance hybrid model generalization to unseen environmental and genetic perturbation conditions

**Fig. 11:**
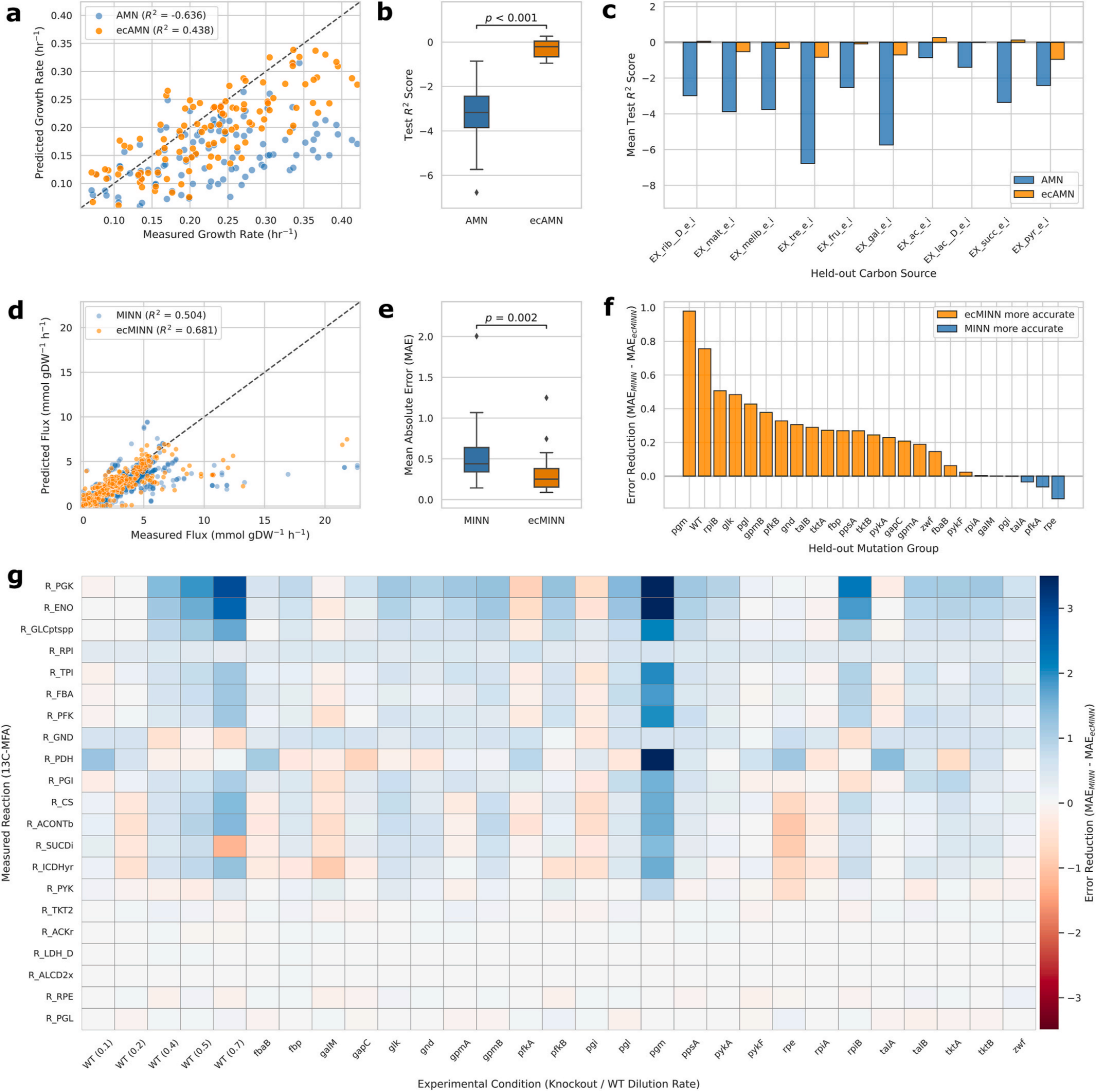
**Comparative cross-validation of standard and enzyme-constrained hybrid models**. **a–c** Leave-One-Substrate-Out (LOSO) cross-validation evaluating the extrapolation performance and model generalization under unseen substrate conditions. **a** Parity plot comparing measured versus predicted growth rates globally for AMN (blue) and ecAMN (orange). **b** Boxplot illustrating the distribution of test R^2^ scores across all held-out substrates. **c** Grouped bar chart detailing the mean test R^2^ scores for each specific held-out carbon source. **d–f** Leave-One-Mutant-Out (LOMO) cross-validation evaluating the generalization performance for flux predictions on held-out mutant strains. **d** Parity plot of measured versus predicted flux values globally for MINN (blue) and ecMINN (orange). **e** Boxplot comparing the Mean Absolute Error (MAE) distributions between the two models, reporting effect sizes and significance. **f** Bar chart mapping the absolute error reduction (MAE_MINN_ - MAE_ecMINN_) for individually held-out mutation groups. Orange bars indicate mutant sets where ecMINN was more accurate, while blue bars signify mutant sets where the baseline MINN had lower error. **g** A diverging heatmap detailing the reaction-specific error reduction across all 29 experimental conditions (5 wild-type dilution rates and 24 single-gene knockouts). The y-axis represents the central carbon metabolism reactions. Blue cells indicate predictions where ecMINN achieved higher accuracy than MINN, visually pinpointing the specific metabolic reactions where enzyme constraints successfully reduce prediction error.

To evaluate model generalization under unseen scenarios, we compared the enzyme-constrained models against their standard counterparts using Leave-One-Substrate-Out (LOSO) and Leave-One-Mutant-Out (LOMO) cross-validation frameworks. Under LOSO evaluation, the ecAMN model demonstrated a substantial improvement in extrapolating growth rates on unseen carbon sources, successfully capturing growth dynamics where the standard AMN model failed to generalize (Fig. 11a). Globally, the enzyme-constrained model attained a positive predictive capability (R^2^ = 0.438), whereas the unconstrained network produced entirely invalid extrapolations that indicate out-of-sample predictions mathematically worse than a simple mean baseline (R^2^ = −0.636). The distribution of test scores R^2^ across substrates (Fig. 11b) confirmed that the ecAMN model consistently outperformed the baseline in generalizing to novel environments (Mann-Whitney *U* test, *p* < 0.001; Cliff's Delta = 0.980; 95% CI of mean difference = [2.029, 4.235]). This enhanced predictive reliability was evident across almost all individual held-out carbon sources (Fig. 11c).

Similarly, LOMO cross-validation revealed that the ecMINN (F*) model yielded more accurate flux predictions for held-out genetic perturbations compared to the baseline MINN (P–F) model (Fig. 11d). The enzyme-constrained formulation successfully captured a better proportion of the global flux variance (ecMINN R^2^ = 0.681 vs. MINN R^2^ = 0.504). Furthermore, the ecMINN model achieved significantly lower (Mann-Whitney *U* test, *p* = 0.002; Cliff's Delta = −0.408; 95% CI of MAE reduction = [0.019, 0.385]) overall prediction errors (Fig. 11e). An analysis of the mutant-specific predictions demonstrated that incorporating enzyme constraints successfully reduced prediction errors across the vast majority of the held-out mutation groups (Fig. 11f). To provide detailed understanding of these improvements, we compared the absolute errors of individual reaction fluxes directly against the experimentally measured ^13^C-MFA targets across all 29 conditions (Fig. 11g). This analysis reveals exactly where the mechanistic constraints exert their influence. For instance, when simulating a *pgm* knockout, the MINN model struggles to accurately predict the flux of other central carbon metabolism reactions due to unconstrained flux rerouting. By enforcing proteome allocation limits, the ecMINN model prevents these unrealistic flux distributions, yielding substantial error reductions across multiple core reactions.

## 4 Discussion

We introduced the EINN framework to address the limitations of current hybrid models by embedding ecGEMs directly into a differentiable mechanistic core. The primary motivation for this integration is to replace the unbounded solution spaces of conventional GEMs with solution spaces that respect the finite catalytic capacity of the proteome. A key factor enabling this integration is the inherent structural compatibility between the GECKO [26] formalism and both the AMN [14] and MINN [15] neural architectures regarding reaction reversibility. Standard flux balance analysis typically handles reversible reactions within a single variable range (negative to positive) [1]; however, differentiable mechanistic layers like AMN and MINN rely on non-negative solvers (e.g., ReLU activations) that necessitate the conversion of all reversible reactions into pairs of irreversible, unidirectional fluxes. While standard GEMs require preprocessing steps to split reversible reactions into forward and reverse components to satisfy this requirement, the GECKO framework naturally enforces this duplication as a fundamental design principle [26]. In the GECKO toolbox, reversible reactions are intrinsically split to account for the distinct enzyme costs and *k*_cat_ associated with the forward and reverse directions. Consequently, the GECKO model structure is natively compatible with the non-negative output constraints of the neural network, allowing the ecGEM to be seamlessly embedded as the mechanistic layer via the loss function that relies on the S-matrix (Fig. 2e and f), which in this case is an expanded S-matrix that includes enzymatic constraints (Fig. 2a–d).

Beyond structural compatibility, the integration of enzyme constraints resolves the critical issue of optimization instability inherent to hybrid models. Because neural networks are highly sensitive to random initial weight assignments set by the random seed, evaluating models across multiple random seeds serves as a standard computational diagnostic for algorithmic stability. Under this multi-seed evaluation, the unconstrained AMN architecture exhibited extreme optimization instability, resulting in a multimodal performance distribution where the network frequently converged to mathematically poor local minima (Fig. 8c). Integrating ecGEMs corrects this by acting as a strict mathematical regularizer. As demonstrated by our FVA results, enzyme constraints reduce the median permissible flux range by an order of magnitude (Fig. 5b–f). This constriction prevents the neural network from overfitting to noise by accessing flux solutions that are stoichiometrically feasible but kinetically impossible. Consequently, this reshaping of the loss landscape ensures reliable convergence and drives substantial performance gains such as the consistent achievement of higher predictive accuracy in ecAMN (Fig. 8a) and significantly lower prediction errors across diverse omics integration strategies in ecMINN (Fig. 10a). While the introduction of these constraints fundamentally alters the feasible flux landscape, evidenced by the lack of correlation between MINN and ecMINN solution spaces (Fig. 10c), it strictly enforces biological reality. Although some ecMINN configurations exhibit a nominally higher ***SV*** loss than their MINN counterparts (Table 3), this reflects an expanded penalty space rather than degraded stoichiometry mass balance. Because ecMINN utilizes an expanded S-matrix, its ***SV*** term strictly penalizes violations of enzyme capacity and protein availability alongside basic mass balance. Navigating this stricter multi-constraint landscape can result in marginally higher ***SV*** losses; however, this rigorous constraint enforcement is precisely what prevents overfitting and secures the framework's superior predictive power. Ultimately, the optimal integration strategies (e.g., ecMINN-F*) successfully reconcile these constraints, achieving ***SV*** losses comparable to MINN baselines while cutting absolute prediction errors (MAE, RMSE) by roughly half.

While we understand the importance of leveraging multi-omics data in this modern day, our evaluation of data integration strategies suggests that the method of omics integration is more critical in the context of hybrid models rather than the total number of omics data, as demonstrated in the ecMINN models performance (Table 3, Fig. 10b). The superior performance of the Intermediate strategy (F*), where proteomics data are treated as physical upper bounds rather than input features, demonstrates that proteomics data yield the highest predictive value when mechanistically integrated, which in this case is used to define the boundaries of the feasible space. Early integration strategy relies on the neural network to implicitly learn the relationship between protein abundance and flux, whereas mechanistic integration explicitly enforces this relationship via the expanded structure of the ecGEM. Furthermore, our results reveal a saturation point in data dimensionality. Once the solution space was bounded by the proteome via the F* strategy, the addition of transcriptomic features only give rise to a small numerical performance gain.

The EINN framework's ability to capture emergent physiological phenomena stems fundamentally from its upgraded mechanistic core. As demonstrated in Fig. 7, standard conventional GEMs exhibit linear, unbounded responses to nutrient availability, whereas the embedded ecGEM components intrinsically recapitulate the non-linear saturation kinetics and acetate switch characteristic of *E. coli* overflow metabolism. By integrating this physically realistic core, the hybrid model is prevented from simply curve-fitting experimental data; instead, the neural network is forced to navigate fundamental resource-allocation trade-offs. This capacity for emergent reasoning is clearly evidenced by the ecAMN-reservoir's performance on carbon utilization rules (Fig. 9b). Without being explicitly trained on carbon source substrate uptake hierarchies, the enzyme-constrained model inferred correct single-source uptake patterns and significantly improved adherence to co-utilization logic compared to the unconstrained baseline. Our detailed breakdown of the prediction records (Supplementary Information 3) highlights where standard stoichiometric layers fail and where enzyme constraints correct them. The standard AMN reservoir's reliance on purely stoichiometric flux optimization inherently prioritizes high-yield glycolytic sugars (Group A) to maximize biomass without consideration for enzymatic costs, consequently dropping lower-yielding Group B organic acids. However, while processing Group A sugars yields high biomass stoichiometrically, doing so requires substantial allocation of cellular machinery to upper glycolysis. Organic acids (Group B) bypass these protein-heavy upper-glycolytic pathways by feeding directly into lower glycolysis and the TCA cycle. By imposing an explicit enzyme capacity limit, ecAMN accurately reflects these cellular crowding constraints. It recognizes that co-utilizing or switching to Group B substrates optimizes biomass production per unit of protein mass allocated, successfully correcting the false negatives produced by the unconstrained model.

Beyond improving training stability and capturing steady-state phenotypic rules, the EINN framework demonstrates a crucial capacity for out-of-distribution generalization, as evidenced by our Leave-One-Substrate-Out (LOSO) and Leave-One-Mutant-Out (LOMO) evaluations (Fig. 11). Standard neural-mechanistic models often struggle to extrapolate outside their specific training domains, which was clearly illustrated when the unconstrained AMN model failed entirely to predict growth on unseen carbon sources (R^2^ = −0.636). By explicitly enforcing enzyme capacity boundaries, the ecAMN framework successfully maintained positive predictive capability in unseen nutritional environments. Similarly, the LOMO evaluation revealed that ecMINN significantly reduces flux prediction errors for unseen genetic perturbations. Under conditions that induce severe metabolic bottlenecks, such as a *pgm* gene knockout, the baseline MINN model suffers from elevated prediction errors across central carbon metabolism. By enforcing strict kinetic boundaries, the enzyme-constrained layer restricts the network to more accurate flux distributions, yielding substantial error reductions across multiple core reactions. Furthermore, ecMINN significantly reduced prediction errors across wild-type sets (Fig. 11f), with particularly notable improvements at high dilution rates (Fig. 11g). This improvement at high dilution rates (conditions known to induce overflow metabolism) demonstrates that ecMINN successfully inherits the underlying ecGEM's capacity to capture complex physiological phenomena that conventional GEMs fundamentally miss. Consequently, grounding the neural network in ecGEMs ensures that the resulting hybrid models produce biologically accurate flux distributions even when predicting the behavior of uncharacterized mutants or novel environments.

To contextualize the performance of the EINN framework models, it is important to clarify our rationale for benchmarking specifically against conventional hybrid models (AMN and MINN) rather than traditional, purely data-driven machine learning algorithms. This scoping boundary relies on the foundational benchmarking exhaustively conducted during the development of the original AMN model [14]. Previous analyses demonstrated that traditional “black-box” artificial neural networks suffer from the curse of dimensionality, requiring training sets orders of magnitude larger (such as 500,000 labeled data points) to reach loss values comparable to hybrid models trained on merely 1000 data points. Furthermore, while standard machine learning algorithms can sometimes fit experimental datasets with acceptable regression performance, they inherently fail to learn underlying mechanistic rules, meaning their outputs cannot guarantee thermodynamic or mass-balance feasibility. Because the fundamental necessity and superiority of hybrid architectures over pure machine learning for valid metabolic flux prediction is already well-established in the literature, our study specifically scopes its evaluation to the next sequential challenge: demonstrating that embedding explicit enzyme capacity constraints (ecGEMs) successfully resolves the biological limitations of standard stoichiometric layers (GEMs) within this hybrid paradigm.

Despite these advances, we acknowledge several limitations of this new proposed conceptual framework. First, the demonstrated results must be interpreted within the constraints of the steady-state assumption inherent to FBA. We excluded dynamic phenomena such as diauxic shifts from our rule-based evaluation because steady-state models cannot represent time-dependent switching without temporal discretization [[45], [46], [47]]. Second, the accuracy of enzyme-constrained formulations remains sensitive to the quality of turnover numbers *k*_cat_. [10,48]. While we mitigated data gaps using deep-learning predictions [30,49], the uncertainty between in vitro and in vivo kinetics remains a challenge [11,50]. Third, we also acknowledge that relying on a static *P*_tot_ parameter, during ecGEM construction, across diverse environmental states represents an engineering approximation of a highly dynamic biological reality, as the experimental availability of condition-specific proteome bounds is often limited. By only utilizing single *P*_tot_ value approximation (following the design principles of the GECKO 3.0 protocol to use approximation value for unknown parameters), the EINN framework models prioritizes generalizable predictive performance over strict condition-specific mechanistic perfection. As demonstrated by our sensitivity analysis where we tested various *P*_tot_ values (Supplementary Information 4), the hybrid architecture is resilient to this simplification as the neural network acts as a compensatory buffer, learning a generalized mapping that respects overarching enzymatic boundaries without strictly overfitting to the exact value of the static *P*_tot_ parameter constraint. Finally, we note that the integration of enzyme constraints introduces a practical computational limitation. The structural expansion of the S-matrix, which must account for thousands of enzyme usage and protein pool pseudo-reactions within the differentiable mechanistic loss function, results in substantially longer training times for EINN models compared to unconstrained baselines (Supplementary Information 5). However, because these hybrid models are primarily utilized in fundamental systems biology and metabolic engineering—fields that prioritize physiological realism and predictive accuracy over the rapid inference times required in fields such as medical and clinical diagnostics—this increased computational cost is an acceptable trade-off and does not significantly hinder their practical application.

In conclusion, our proposed EINN framework demonstrates improvement of metabolic prediction accuracy of hybrid models is possible by tightening the mechanistic boundaries within which neural networks operate. By unifying the adaptive capacity of neural networks with the biophysical realism of enzyme-constrained models, EINN provides a robust, stable, and reliable platform for understanding complex phenotypes and engineering microbial cell factories. Even though we only demonstrate EINN framework models that rely on GECKO 3.0 to constrain the model enzymatically, further research can explore instances of EINN framework models that rely on other enzyme-constraining methods such as CORAL[22], sMOMENT [13], MOMENT [23], ECMpy [24], and FBAwMC [25]. Looking ahead, the versatility of this framework invites integration with even more rigorous biophysical constraints. Future studies could explore the incorporation of thermodynamic constraints [51] or combined thermodynamic-enzyme constraints [52,53] to further reduce the solution space and eliminate thermodynamically infeasible fluxes. Furthermore, to transcend the limitations of steady-state assumptions inherent in standard FBA, this framework could be adapted for dynamic FBA (dFBA) [45]. Such an expansion would necessitate coupling the mechanistic layer with sequence-optimized architectures, such as Long Short-Term Memory (LSTM) networks [54] or Gated Recurrent Units (GRUs) [55], to enable the precise modeling of time-resolved metabolic shifts.

## Data availability

The GEMs used in this study were obtained from the BiGG Models database (http://bigg.ucsd.edu/). This work also makes use of the original datasets and source code from AMN (https://github.com/brsynth/amn_release) and MINN frameworks (https://github.com/gabrieletaz/MINN). Multi-omics datasets for *E. coli* were obtained from the Keio University *E. coli* Omics Database (https://ecoli.iab.keio.ac.jp/). All processed data, model configurations, and scripts necessary to reproduce the results of this study are available in the accompanying repository (see “Code availability”). Additional data and materials are available from the corresponding authors upon reasonable request.

## Code availability

All scripts and datasets used to generate the results reported in this study are provided in a publicly documented repository. A citable, archived version of the repository associated with this work is available via Zenodo at https://doi.org/10.5281/zenodo.18347066. For access to the most up-to-date versions of the code, as well as to report issues or interact with the authors, the repository is also maintained on GitHub at https://github.com/raysteven/EINN.

## CRediT authorship contribution statement

**Ray Steven:** Conceptualization, Formal analysis, Investigation, Methodology, Software, Visualization, Writing – original draft. **Kai Kimata:** Writing – review & editing. **Denis Chegodaev:** Writing – review & editing. **Lilies Handayani:** Writing – review & editing. **Kenji Satou:** Supervision, Writing – review & editing.

## Declaration of competing interest

The authors declare that they have no known competing financial interests or personal relationships that could have appeared to influence the work reported in this paper.
