# Supplementary material for: EINN: An enzyme-informed neural network guided by an enzyme-constrained genome-scale metabolic model
Source: Synth Syst Biotechnol. 2026 Aug 6;17:66–84. doi: 10.1016/j.synbio.2026.07.009 (PMC13474246; doi:10.1016/j.synbio.2026.07.009)
Supplement: Supplementary file 1 [file mmc1.docx]

# Supplementary Information 1

## Metabolic Pathway Classification

To examine enzyme distribution patterns, metabolic pathways from KEGG were systematically mapped to broader functional categories. This assignment followed the established framework of Bar-Even et al. (2011), which organizes pathways into three principal metabolic classes. Given the limited representation of secondary metabolic enzymes in the dataset, pathways for secondary and intermediate metabolism were consolidated into a single category. The resulting classification is presented in Table S1, and its impact on enzyme distribution is illustrated in Figures 5C, 5D, 5G, 5H.

**Table S1.** Classification of KEGG Pathways into Metabolic Groups.

| **Pathway** | **Classification** |
| --- | --- |
| 2-Oxocarboxylic acid metabolism | CE |
| ABC transporters | ISO |
| Alanine, aspartate and glutamate metabolism | AFN |
| Amino sugar and nucleotide sugar metabolism | AFN |
| Aminoacyl-tRNA biosynthesis | ISO |
| Aminobenzoate degradation | AFN |
| Arginine and proline metabolism | AFN |
| Arginine biosynthesis | AFN |
| Ascorbate and aldarate metabolism | CE |
| Bacterial chemotaxis | ISO |
| Benzoate degradation | AFN |
| Biofilm formation - Escherichia coli | AFN |
| Biosynthesis of amino acids | AFN |
| Biosynthesis of cofactors | AFN |
| Biosynthesis of nucleotide sugars | AFN |
| Biosynthesis of siderophore group nonribosomal peptides | ISO |
| Biosynthesis of unsaturated fatty acids | AFN |
| Biosynthesis of various nucleotide sugars | AFN |
| Biotin metabolism | ISO |
| Butanoate metabolism | CE |
| C5-Branched dibasic acid metabolism | AFN |
| Carbon metabolism | CE |
| Cationic antimicrobial peptide (CAMP) resistance | AFN |
| Citrate cycle (TCA cycle) | CE |
| Cobalamin transport and metabolism | AFN |
| Cyanoamino acid metabolism | AFN |
| Cysteine and methionine metabolism | AFN |
| D-Amino acid metabolism | AFN |
| Degradation of aromatic compounds | AFN |
| Ether lipid metabolism | AFN |
| Exopolysaccharide biosynthesis | AFN |
| Fatty acid biosynthesis | AFN |
| Fatty acid degradation | AFN |
| Fatty acid metabolism | AFN |
| Flagellar assembly | ISO |
| Folate biosynthesis | ISO |
| Folate transport and metabolism | AFN |
| Fructose and mannose metabolism | AFN |
| Galactose metabolism | CE |
| Glutathione metabolism | AFN |
| Glycerolipid metabolism | AFN |
| Glycine, serine and threonine metabolism | AFN |
| Glycolysis / Gluconeogenesis | CE |
| Glyoxylate and dicarboxylate metabolism | CE |
| Histidine metabolism | AFN |
| Inositol phosphate metabolism | CE |
| Lipoic acid metabolism | AFN |
| Lipopolysaccharide biosynthesis | AFN |
| Lysine biosynthesis | AFN |
| Lysine degradation | AFN |
| Microbial metabolism in diverse environments | CE |
| Nicotinate and nicotinamide metabolism | AFN |
| Nitrogen metabolism | CE |
| Nucleotide metabolism | AFN |
| O-Antigen repeat unit biosynthesis | AFN |
| One carbon pool by folate | AFN |
| Oxidative phosphorylation | CE |
| Pantothenate and CoA biosynthesis | AFN |
| Pentose and glucuronate interconversions | CE |
| Pentose phosphate pathway | CE |
| Peptidoglycan biosynthesis | AFN |
| Phenylalanine metabolism | AFN |
| Phenylalanine, tyrosine and tryptophan biosynthesis | AFN |
| Phosphonate and phosphinate metabolism | AFN |
| Phosphotransferase system (PTS) | CE |
| Porphyrin metabolism | ISO |
| Propanoate metabolism | CE |
| Purine metabolism | AFN |
| Pyrimidine metabolism | AFN |
| Pyruvate metabolism | CE |
| Quorum sensing | ISO |
| RNA degradation | CE |
| Riboflavin metabolism | ISO |
| Selenocompound metabolism | AFN |
| Starch and sucrose metabolism | AFN |
| Sulfur metabolism | AFN |
| Sulfur relay system | ISO |
| Taurine and hypotaurine metabolism | AFN |
| Terpenoid backbone biosynthesis | ISO |
| Thiamine metabolism | ISO |
| Tryptophan metabolism | AFN |
| Two-component system | CE |
| Tyrosine metabolism | AFN |
| Ubiquinone and other terpenoid-quinone biosynthesis | ISO |
| Valine, leucine and isoleucine biosynthesis | AFN |
| Valine, leucine and isoleucine degradation | AFN |
| Vitamin B6 metabolism | ISO |
| beta-Alanine metabolism | AFN |
| beta-Lactam resistance | ISO |

CE: Carbohydrate and energy primary metabolism, AFN: Amino acid, fatty acid, and nucleotide primary metabolism , ISO: Intermediate and secondary metabolism.

# Supplementary Information 2

## Flux Reconciliation via Euclidean Projection

### 1. Rationale for Flux Projection

The experimental flux distributions obtained from ^13^C-MFA (Ishii et al., 2007) were originally estimated using a simplified core metabolic model. When mapped onto the iAF1260 and eciAF1260 metabolic network, these fluxes are often stoichiometrically inconsistent because the core model does not account for the full requirement of biomass precursors and secondary metabolic constraints inherent in the genome-scale reconstruction.

To resolve this, we applied a Euclidean projection method. This approach reconciles experimental measurements with newer GEMs by finding the closest feasible flux distribution within the high-dimensional solution space of the iAF1260 and eciAF1260 network.

### 2. Mathematical Formulation

To resolve these mechanistic conflicts, we implemented a single-step optimization procedure to project experimental fluxes (*v_exp_*) onto the feasible solution space of the GEM while directly incorporating experimental constraints. The reconciliation is formulated as a convex quadratic programming (QP) problem where the objective is to find a feasible flux vector *v* that minimizes the sum of squared differences (Euclidean distance) for the subset of measured reactions 𝒨, subject to stoichiometric and experimental constraints:

<https://www.codecogs.com/eqnedit.php?latex=%5Cbegin%7Bequation%7D%20%5Cbegin%7Baligned%7D%5Bb%5D%20%5Ctext%7Bminimize%7D%20%5Cquad%20%26%20%5Csum_%7Bi%20%5Cin%20%5Cmathcal%7BM%7D%7D%20(v_i%20-%20v_%7Bexp%2Ci%7D)%5E2%20%5C%5C%5Ctext%7Bsubject%20to%7D%20%5Cquad%20%26%20S%20%5Ccdot%20v%20%3D%200%20%5C%5C%26%20lb_j%20%5Cleq%20v_j%20%5Cleq%20ub_j%2C%20%5Cquad%20%5Cforall%20j%20%5Cin%20%5C%7B1%2C%20%5Cdots%2C%20n%5C%7D%20%5C%5C%26%20v_%7Bbiomass%7D%20%3D%20%5Cmu_%7Bexp%7D%5Cend%7Baligned%7D%20%5Chspace%7B1.5cm%7D%20%5Ctag%7B2%7D%20%5Cend%7Bequation%7D#0>

where *S* is the stoichiometric matrix of the genome-scale model, and *lb_j_*, *ub_j_* represent the reaction bounds. The growth rate is constrained to the experimentally observed dilution rate (*μ_exp_*) to ensure biological consistency.

### 3. Computational Implementation

The reconciliation pipeline was implemented in Python 3.9 using the COBRApy library for model handling and CVXPY for convex optimization. We utilized the OSQP (Operator Splitting Quadratic Program) solver for its efficiency in handling quadratic objectives, with the SCS solver as a fallback. Experimental fluxes, originally reported as percentages of glucose uptake, were converted to absolute molar fluxes (mmol.gDW^-1^.h^-1^) using the measured specific glucose uptake rate (*q_glc_*) of each sample before the projection process.

# Supplementary Information 3

## Detailed Breakdown of Carbon Source Utilization Predictions

Table S2 presents a case-by-case breakdown of specific carbon uptake conditions mispredicted by the standard AMN-reservoir model but successfully resolved by the enzyme-constrained ecAMN-reservoir model. As observed in the data, the unconstrained AMN-reservoir consistently fails to predict phenotypes involving the uptake of Group B carbon sources (such as acetate, succinate, and pyruvate). In single-source environments, the AMN-reservoir model drops the Group B substrates entirely, predicting no uptake. Furthermore, in mixed environments containing both Group A (e.g., maltose) and Group B sources, the AMN-reservoir model fails to predict co-utilization, artificially restricting uptake to solely the Group A carbon source. By imposing explicit enzyme capacity limits, the ecAMN-reservoir model successfully resolves these specific blind spots. It correctly captures these physiological phenotypes, accurately predicting the uptake of single Group B sources and the necessary co-utilization strategies required to optimize biomass production under finite protein allocation constraints.

**Table S2.** Comparison of carbon source uptake predictions between standard AMN-reservoir and ecAMN-reservoir models.

| **Carbon Utilization Uptake Phenotype** | **Ground Truth** | | **AMN-reservoir** | | | **ecAMN-reservoir** | | |
| --- | --- | --- | --- | --- | --- | --- | --- | --- |
|  | **Group A Carbon Source**  **Uptake** | **Group B  Carbon Sources**  **Uptake** | **Predicted**  **Group A Carbon Source**  **Uptake** | **Predicted**  **Group B  Carbon Source**  **Uptake** | **Prediction Correct?** | **Predicted**  **Group A Carbon Source**  **Uptake** | **Predicted**  **Group B  Carbon Source**  **Uptake** | **Prediction Correct?** |
| Single Source | - | EX_ac_e_i | - | - | FALSE | - | EX_ac_e_i | TRUE |
| Single Source | - | EX_succ_e_i | - | - | FALSE | - | EX_succ_e_i | TRUE |
| Co-utilization | - | EX_pyr_e_i, EX_succ_e_i | - | EX_pyr_e_i | FALSE | - | EX_pyr_e_i, EX_succ_e_i | TRUE |
| Co-utilization | EX_malt_e_i | EX_ac_e_i | EX_malt_e_i | - | FALSE | EX_malt_e_i | EX_ac_e_i | TRUE |
| Co-utilization | EX_gal_e_i | EX_succ_e_i | EX_gal_e_i | - | FALSE | EX_gal_e_i | EX_succ_e_i | TRUE |
| Co-utilization | - | EX_ac_e_i, EX_lac__D_e_i | - | EX_lac__D_e_i | FALSE | - | EX_ac_e_i, EX_lac__D_e_i | TRUE |
| Co-utilization | EX_tre_e_i | EX_ac_e_i | EX_tre_e_i | - | FALSE | EX_tre_e_i | EX_ac_e_i | TRUE |
| Co-utilization | EX_rib__D_e_i | EX_ac_e_i | EX_rib__D_e_i | - | FALSE | EX_rib__D_e_i | EX_ac_e_i | TRUE |
| Co-utilization | EX_tre_e_i | EX_succ_e_i | EX_tre_e_i | - | FALSE | EX_tre_e_i | EX_succ_e_i | TRUE |
| Co-utilization | - | EX_succ_e_i, EX_lac__D_e_i | - | EX_lac__D_e_i | FALSE | - | EX_succ_e_i, EX_lac__D_e_i | TRUE |
| Co-utilization | EX_melib_e_i | EX_succ_e_i | EX_melib_e_i | - | FALSE | EX_melib_e_i | EX_succ_e_i | TRUE |
| Co-utilization | EX_melib_e_i | EX_ac_e_i, EX_succ_e_i | EX_melib_e_i | - | FALSE | EX_melib_e_i | EX_ac_e_i, EX_succ_e_i | TRUE |
| Co-utilization | EX_rib__D_e_i | EX_ac_e_i, EX_succ_e_i | EX_rib__D_e_i | - | FALSE | EX_rib__D_e_i | EX_ac_e_i, EX_succ_e_i | TRUE |

# Supplementary Information 4

## Protein Pool Sensitivity Analysis

To evaluate the robust performance of the architecture under changing physiological constraints, a sensitivity analysis was performed by perturbing the total protein mass (P_tot_) parameters across a range of -20% to +20% relative to the baseline model (Fig. S1). A global statistical assessment via the Friedman test revealed no significant difference (*p* > 0.05) in the per-sample absolute error distributions among the five distinct configurations (*p* = 0.1619). This lack of statistical divergence strongly indicates that the upstream neural layer successfully functions as a metabolic buffer, absorbing variations in P_tot_ levels and maintaining stable, highly accurate growth rate predictions without necessitating a hard structural recalibration of the core mechanistic model.

**Figure S1**. Sensitivity analysis of model absolute errors across variations in total protein mass (P_tot_). (a) Violin plots detailing the distribution of individual sample absolute errors (the absolute difference between predicted and experimentally measured growth rates) for the -20%, -10%, Base, +10%, and +20% P_tot_ configurations. Internal horizontal dashed and dotted lines indicate the median and interquartile ranges, respectively. (b) Sample-wise error parity plot tracking the absolute error agreement between the extreme variant models (-20% shown as blue circles; +20% shown as orange squares) and the unperturbed Base model. The diagonal dashed line represents the 1:1 line of perfect error agreement.

# Supplementary Information 5

# Training Runtime

To assess the computational overhead introduced by embedding enzyme constraints, we compared the mean training runtimes of the proposed EINN variants (ecAMN, ecAMN-reservoir, and ecMINN) against their unconstrained baselines (AMN, AMN-reservoir, and MINN). All recorded runtime was during the Leave-One-Out Cross-Validation (LOOCV) experiment. As detailed in Table S3, the incorporation of enzyme constraints consistently increases the computational time required for training. This increase in computational cost is an expected architectural trade-off as it directly reflects the substantially larger dimensions of the expanded stoichiometric matrices in ecGEMs, which introduce thousands of additional pseudo-reactions and pseudo-metabolites. Consequently, the differentiable solvers require more time to navigate this expanded, highly restricted optimization landscape to strictly enforce proteomic and enzymatic capacity constraints during the learning process.

**Table S3.** Mean training runtimes for standard and enzyme-constrained hybrid models.

| **Model** | **Mean Training Runtime (s)** | **Training Configuration** |
| --- | --- | --- |
| AMN | 34.35 | LOOCV |
| ecAMN | 288.69 | LOOCV |
| AMN-reservoir | 1458.33 | LOOCV |
| ecAMN-reservoir | 5004.73 | LOOCV |
| MINN | 722.33 | LOOCV |
| ecMINN | 1279.22 | LOOCV |

# References

Bar-Even, A. *et al*. The Moderately Efficient Enzyme: Evolutionary and Physicochemical Trends Shaping Enzyme Parameters. *Biochemistry* **50**, 4402–4410 (2011).
